# Disability Transitions and Health Expectancies among Adults 45 Years and Older in Malawi: A Cohort-Based Model

**DOI:** 10.1371/journal.pmed.1001435

**Published:** 2013-05-07

**Authors:** Collin F. Payne, James Mkandawire, Hans-Peter Kohler

**Affiliations:** 1 Graduate Group in Demography, University of Pennsylvania, Philadelphia, Pennsylvania, United States of America; 2 Population Studies Center, University of Pennsylvania, Philadelphia, Pennsylvania, United States of America; 3 Invest in Knowledge, Zomba, Malawi; 4 Department of Sociology, University of Pennsylvania, Philadelphia, Pennsylvania, United States of America; London School of Hygiene and Tropical Medicine, United Kingdom

## Abstract

**Background:**

Falling fertility and increasing life expectancy contribute to a growing elderly population in sub-Saharan Africa (SSA); by 2060, persons aged 45 y and older are projected to be 25% of SSA's population, up from 10% in 2010. Aging in SSA is associated with unique challenges because of poverty and inadequate social supports. However, despite its importance for understanding the consequences of population aging, the evidence about the prevalence of disabilities and functional limitations due to poor physical health among older adults in SSA continues to be very limited.

**Methods and Findings:**

Participants came from 2006, 2008, and 2010 waves of the Malawi Longitudinal Survey of Families and Health, a study of the rural population in Malawi. We investigate how poor physical health results in functional limitations that limit the day-to-day activities of individuals in domains relevant to this subsistence-agriculture context. These disabilities were parameterized based on questions from the SF-12 questionnaire about limitations in daily living activities. We estimated age-specific patterns of functional limitations and the transitions over time between different disability states using a discrete-time hazard model. The estimated transition rates were then used to calculate the first (to our knowledge) microdata-based health expectancies calculated for SSA. The risks of experiencing functional limitations due to poor physical health are high in this population, and the onset of disabilities happens early in life. Our analyses show that 45-y-old women can expect to spend 58% (95% CI, 55%–64%) of their remaining 28 y of life (95% CI, 25.7–33.5) with functional limitations; 45-y-old men can expect to live 41% (95% CI, 35%–46%) of their remaining 25.4 y (95% CI, 23.3–28.8) with such limitations. Disabilities related to functional limitations are shown to have a substantial negative effect on individuals' labor activities, and are negatively related to subjective well-being.

**Conclusions:**

Individuals in this population experience a lengthy struggle with disabling conditions in adulthood, with high probabilities of remitting and relapsing between states of functional limitation. Given the strong association of disabilities with work efforts and subjective well-being, this research suggests that current national health policies and international donor-funded health programs in SSA inadequately target the physical health of mature and older adults.

*Please see later in the article for the Editors' Summary*

## Abstract 2

Collin Payne and colleagues investigated development of disabilities and years expected to live with disabilities in participants 45 years and older participating in the Malawi Longitudinal Survey of Families and Health.

*Please see later in the article for the Editors' Summary*

## Editors' Summary

**Background:**

The population of the world is getting older. In almost every country, the over-60 age group is growing faster than any other age group. In 2000, globally, there were about 605 million people aged 60 years or more; by 2050, 2 billion people will be in this age group. Much of this increase in the elderly population will be in low-income countries. In sub-Saharan Africa, for example, 10% of the population is currently aged 45 years or more, but by 2060, a quarter of the population will be so-called mature adults. In all countries, population aging is the result of women having fewer children (falling fertility) and people living longer (increasing life expectancy). Thus, population aging is a demographic transition, a change in birth and death rates. In low- and middle-income countries, population aging is occurring in parallel with an “epidemiological transition,” a shift from communicable (infectious) diseases to non-communicable diseases (for example, heart disease) as the primary causes of illness and death.

**Why Was This Study Done?:**

Both the demographic and the epidemiological transition have public health implications for low-income countries. Good health is important for the independence and economic productivity of older people. Productive older people can help younger populations financially and physically, and help compensate for the limitations experienced by younger populations infected with HIV. Also, low-income countries lack social safety nets, so disabled older adults can be a burden on younger populations. Thus, the health of older individuals is important to the well-being of people of all ages. As populations age, low-income countries will need to invest in health care for mature and elderly adults and in disease prevention programs to prevent or delay the onset of non-communicable diseases, which can limit normal daily activities by causing disabilities. Before providing these services, national policy makers need to know the proportion of their population with disabilities, the functional limitations caused by poor physical health, and the health expectancies (the number of years a person can expect to be in good health) of older people in their country. In this cohort modeling study, the researchers estimate health expectancies and transition rates between different levels of disability among mature adults in Malawi, one of the world's poorest countries, using data collected by the Malawi Longitudinal Survey of Families and Health (MLSFH) on economic, social, and health conditions in a rural population. Because Malawi has shorter life expectancies and earlier onset of disability than wealthier countries, the authors considered individuals aged 45 and older as mature adults at risk for disability.

**What Did the Researchers Do and Find?:**

The researchers categorized the participants in the 2006, 2008, and 2010 waves of the MLSFH into three levels of functional limitation (healthy, moderately limited, and severely limited) based on answers to questions in the SF-12 health survey questionnaire that ask about disabilities that limit daily activities that rural Malawians perform. The researchers estimated age–gender patterns of functional limitations and transition rates between different disability states using a discrete-time hazard model, and health expectancies by running a microsimulation to model the aging of synthetic cohorts with various starting ages but the same gender and functional limitation distributions as the study population. These analyses show that the chance of becoming physically disabled rises sharply with age, with 45-year-old women in rural Malawi expected to spend 58% of their estimated remaining 28 years with functional limitations, and 45-year-old men expected to live 41% of their remaining 25.4 years with functional limitations. Also, on average, a 45-year-old woman will spend 2.7 years with moderate functional limitation and 0.6 years with severe functional limitation before she reaches 55; for men the corresponding values are 1.6 and 0.4 years. Around 50% of moderately and 60%–80% of severely limited individuals stated that pain interfered quite a bit or extremely with their normal work during the past four weeks, suggesting that pain treatment may help reduce disability.

**What Do These Findings Mean?:**

These findings suggest that mature adults in rural Malawi will have some degree of disability during much of their remaining lifetime. The risks of experiencing functional limitations are higher and the onset of persistent disabilities happens earlier in Malawi than in more developed contexts—the proportions of remaining life spent with severe limitations at age 45 in Malawi are comparable to those of 80-year-olds in the US. The accuracy of these findings is likely to be affected by assumptions made during modeling and by the quality of the data fed into the models. Nevertheless, these findings suggest that functional limitations, which have a negative effect on the labor activity of individuals, will become more prominent in Malawi (and probably other sub-Saharan countries) as the age composition of populations shifts over the coming years. Older populations in sub-Saharan Africa are not targeted well by health policies and programs at present. Consequently, these findings suggest that policy makers will need to ensure that additional financial resources are provided to improve health-care provision for aging individuals and to lessen the high rates of functional limitation and associated disabilities.

**Additional Information:**

Please access these websites via the online version of this summary at http://dx.doi.org/10.1371/journal.pmed.1001435.

- This study is further discussed in a *PLoS Medicine* Perspective by Andreas Stuck, et al.
- The World Health Organization provides information on many aspects of aging (in several languages); the WHO Study on Global Ageing and Adult Health (SAGE) is compiling longitudinal information on the health and well-being of adult populations and the aging process
- The United Nations Population Fund and HelpAge International publication Ageing in the Twenty-First Century is available
- HelpAge International is an international nongovernmental organization that helps older people claim their rights, challenge discrimination, and overcome poverty, so that they can lead dignified, secure, and healthy lives
- More information on the Malawi Longitudinal Study of Families and Health is available

## Introduction

**Figure 1:**
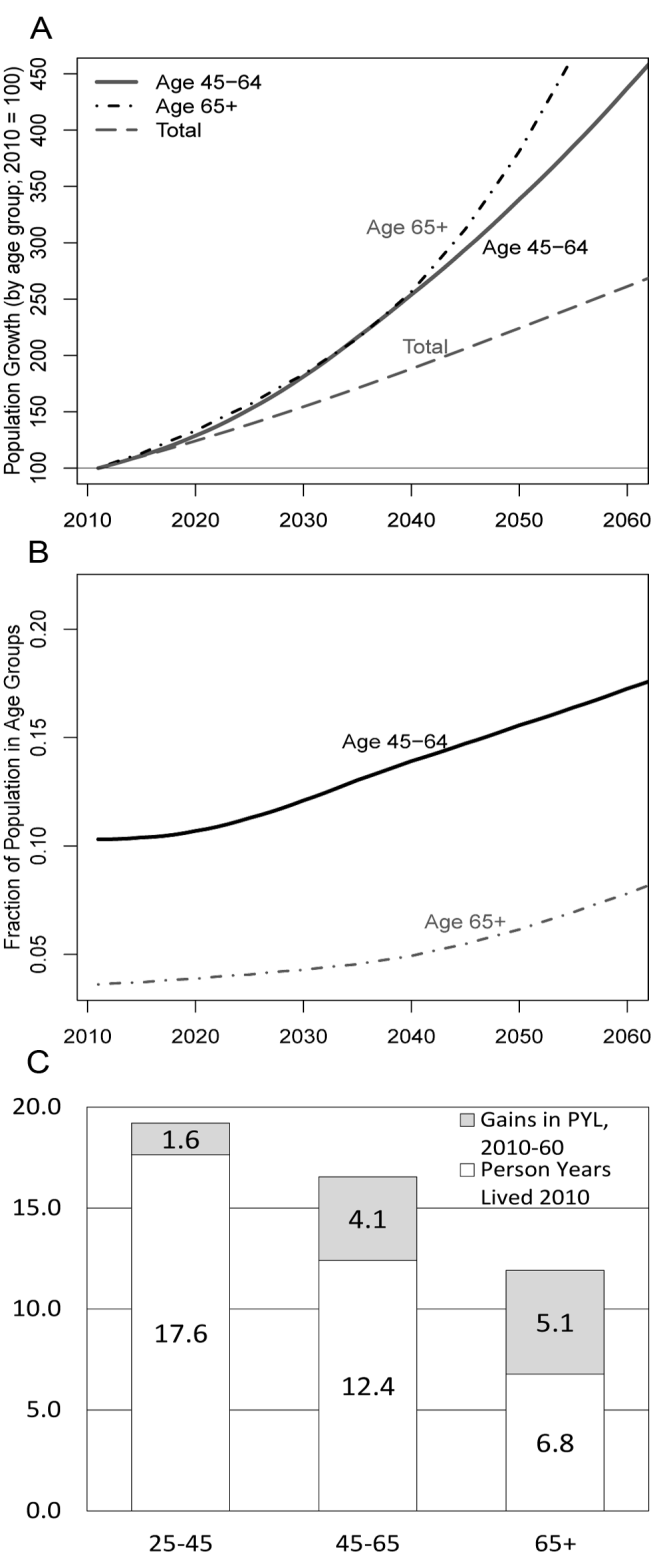
Population growth by age group in SSA 2010–2060, share of total population by age groups, and person-years lived by age group. (A) The projected population growth by age group in SSA, 2010–2060 (2010 = 100). (B) The share of the total SSA population by age group. (C) Person-years lived (PYL) by age group. Source: authors' calculations based on United Nations population projections [5].

While rapid population growth continues to be a major social and policy issue in sub-Saharan Africa (SSA) [1],[2], demographic and epidemiological trends of falling fertility and increasing life expectancy (LE) foreshadow the coming challenge of a growing elderly population in SSA. Because of high levels of morbidity, low levels of economic development, and widespread poverty, aging in SSA will likely be associated with a unique set of demographic and economic challenges [3],[4]. The age group of mature adults (defined here as adults aged 45 y and older) deserves particular attention in this context (Figure 1). The population of mature adults aged 45+ y will expand more rapidly in the next decades than that of any younger 10-y age group in many SSA low-income countries (LICs). By 2060, persons aged 45+ y are projected to be 25% of SSA's population [5], up from 10% in 2010, and the 65+-y population alone is expected to represent more than 5% of the SSA population after 2040 (Figure 1B). Over the next 50 y in SSA LICs such as Malawi, 80% of the additional person-years lived by adults aged 25+ y as a result of increasing LEs will occur among individuals aged 45+ y (Figure 1C): 4.1 additional years, or 38% of the overall adult LE gain, will occur among individuals aged 45–64 y, and 5.1 y, or 47% of the adult LE gain, will occur among individuals aged 65+ y. HIV prevalence among mature adults is currently still relatively low [6], but is expected in increase as younger cohorts with higher HIV prevalence benefit from higher LE as a result of antiretroviral treatment [7].

Intertwined with this demographic transformation of SSA is the epidemiological transition of diseases, in which the primary causes of morbidity and mortality increasingly shift from communicable to noncommunicable diseases. During the period 1990–2010, the disease burden attributable to childhood communicable diseases decreased in central, eastern, and western SSA, both as a proportion of total disease burden and in rank order, while risk factors for some noncommunicable diseases and injury accounted for a larger disease burden in 2010 [8]. SSA regions currently have among the globally highest levels of years lived with disability for men and women after age 45 y [9], with combined levels for women in SSA 54% higher than in Europe (16% for men) and 21% higher than in Asia (16% for men). By 2030, it is estimated that chronic noncommunicable diseases will cause 47% of deaths in Africa, compared with about 27% in 2008, while mortality from communicable diseases is predicted to decline from 53% to 30% [10]. Scholars and policy makers are only beginning to recognize the health and social policy challenges of population aging in SSA LICs [7],[11]–[24], particularly in contexts that have been substantially affected by the HIV/AIDS epidemic.

SSA LICs are characterized by high to very high rates of economic activity across all adult age groups [25]. In Malawi, labor force participation is virtually universal, even among mature adults (98% for ages 50–64 y and 90% for age 65+ y, based on the 2009 Malawi Welfare Monitoring Survey [26]). If low levels of disability among mature adults (age 45+ y) can be ensured, this population can contribute significantly to aggregate economic growth and individual/family well-being during the next decades. Contributions from mature adults are critical: in the absence of widespread institutionalized social security and health insurance programs, they can be providers of intergenerational and intragenerational economic transfers that could ameliorate the consequences of the HIV epidemic and other social/economic crises for family members [27]–[31]. Evidence suggests that chronic and disabling conditions among the mature adult population, resulting from the cumulative effects of poor nutrition and frequent exposure to infectious disease, lead to significant levels of functional limitations in day-to-day activities and a substantial gap between potential and actual economic productivity [15],[18],[19],[32],[33].

Despite its importance for understanding the consequences of population aging and developing adequate policy responses, evidence about the prevalence of disabilities, the level of functional limitations due to poor physical health, and the pattern of health trajectories among older adults in SSA continues to be very limited. For example, while national health sector strategic plans in Malawi and other SSA countries highlight the need for policies to prevent disabilities and ensure access to curative and rehabilitative care among older individuals [34]–[36], there is a dearth of understanding among national and international decision-makers about the magnitude of the aging problem in SSA, the scope of old-age-related health needs, and the trajectories of health and disability at mature and old ages [23],[24],[37]. Evidence from more developed contexts is generally not sufficient for understanding these emerging health issues and health-care needs among the growing aging population in SSA, as interactions among infectious/noninfectious diseases and/or exposure to malnutrition and poverty can result in distinct patterns of and risk factors for poor health and disabilities among mature adults [24],[38].

Our analyses seek to fill some of these gaps in existing knowledge by investigating how the physical health of rural Malawians results in functional limitations, that is, by studying how physical health limits the day-to-day activities of individuals in domains relevant to this subsistence-agriculture context. We estimate age patterns of functional limitations and the transitions over time between different disability states, and calculate health expectancies (HEs). To our knowledge, our estimates are the first microdata-based HEs calculated for SSA. Together, these measures characterize processes of health, aging, and functional limitations and associated disabilities in a rapidly growing but understudied portion of the SSA population, and can provide important insights into the potential gains in well-being and economic productivity arising from investments in the health of and health care for mature and older adults in SSA.

## Methods

### Context

Malawi is an opportune environment for studying epidemiologic and demographic transitions and their implications for the health and well-being of mature adults in SSA. It is one of the poorest countries in the world, ranked 153 of 169 in terms of the human development index [39], with about 15% of its population considered “ultra-poor”, that is, with an estimated food consumption below the minimum level of dietary energy requirement [40]. LE at birth was estimated to be 51 y for men and 55 y for women in 2010, and healthy LE at birth is estimated at 44 y for men and 46 y for women [41]. While the Malawian per capita income is below the SSA average, Malawi is similar to other SSA countries and countries classified as LICs by the World Bank in terms of LE, infant mortality, child malnutrition, access to clean water, literacy, and educational enrollment [42],[43]. In rural areas, where our study population is based, the majority of individuals engage in home production of crops, primarily maize, squash, tomatoes, potatoes, nuts, dark green leafy vegetables, and fruit, complemented by some market activities. While tuberculosis, malaria, and endemic parasites (e.g., soil-transmitted helminths and *Schistosoma mansoni*) have a relatively high prevalence [44],[45], chronic diseases such as hypertension and diabetes and disease risk factors such as tobacco use and alcohol consumption also affected a substantial proportion of mature and older adults in rural areas [15]. Moreover, while HIV/AIDS is widespread, the vast majority of the population—more than 85% of adults aged 15–49 y, and an even higher fraction among adults aged 50 y and over—is HIV-negative [6],[46]. Yet, HIV-negative individuals also confront a high-disease-risk environment characterized by high levels of poverty, episodic malnutrition, poor sanitation, a high prevalence of infectious diseases and endemic parasites, and limited access to health-care facilities. The cumulative load of these pressures may have substantial consequences for health, well-being, and functional limitations that persist throughout the remaining life course [47].

### Data

The Malawi Longitudinal Survey of Families and Health (MLSFH) is a longitudinal study of the rural population in Malawi that monitors the social, economic, and health conditions in one of the world's poorest nations. The study is based in three districts in rural Malawi: Rumphi in the north, Mchinji in the center, and Balaka in the south (Figure S1). While these rural regions are similar in terms of their overall epidemiological, socioeconomic, and subsistence-agriculture characterization [44],[48], the regions reflect some heterogeneity in terms of marriage patterns [49], religious affiliations [50], schooling [51], patrilineal versus matrilineal inheritance and land ownership [52], and HIV prevalence [46],[53]. MLSFH respondents (*N*_2010_≈3,800) are evenly split among the three study locations and clustered in 121 villages. MLSFH rounds were collected in 1998, 2001, 2004, 2006, 2008, and 2010. In 2008, the MLSFH added a sample of about 550 parents of original MLSFH respondents, substantially increasing the population of mature adults (aged 45+ y) in the study. The MLSFH sampling methods and related relevant data collection procedures are described in Text S1. Over 40% of the 2010 MLSFH study population is currently aged 45 y and older, and 12% is 65 y and older. The prevalence of HIV is estimated at 11% among 15- to 49-y-old individuals in Malawi [54], and it has been shown to decline substantially with age at older ages [6]. For example, HIV testing among mature adults (individuals aged 45+ y) in the 2008 MLSFH found an overall HIV prevalence of 3.3%. Most (80.5%) HIV-positive mature adults were younger than age 55 y, and there were only two HIV-positive individuals over age 65 y in the 2008 MLSFH. Though there may be important differences in disability trajectories among HIV-positive and HIV-negative individuals, our analyses do not differentiate by HIV status. This pooling of HIV-positive and HIV-negative individuals in our study is substantively justified, as the HIV prevalence among mature adults is low and HIV/AIDS is only one of the many diseases affecting the mature adult population in contexts such as Malawi [8],[9],[23],[37].

**Figure 2:**
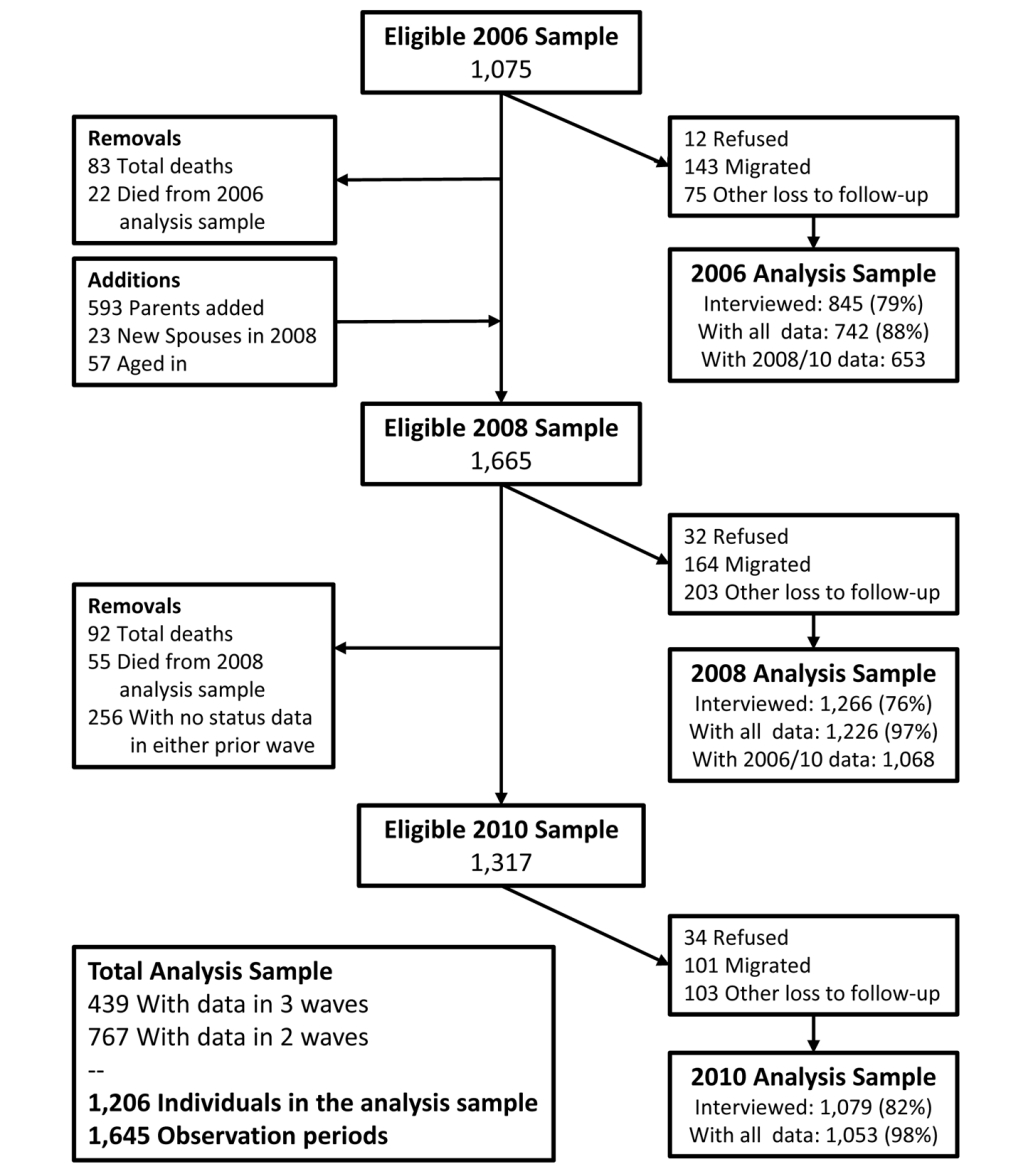
MLSFH sample flow 2006–2010.

Data for the current analyses come from MLSFH waves collected in 2006, 2008, and 2010 that contain the longitudinal health data required for our analyses. Functional limitations and the resulting disability states of individuals are determined from a set of questions on self-reported health and disabilities based on a locally suitable version of the SF-12 health survey [55], a survey instrument for measuring self-reported health that has been validated in SSA and globally. Nonresponse primarily resulted from respondents moving to another area or being temporarily absent; fewer than 3% of respondents refused to be interviewed in each wave. Because of our focus on transitions in disability status among mature adults over time, our analysis sample was restricted to individuals age 45 y or older who participated in at least two MLSFH rounds, or were interviewed once but died between MLSFH rounds. Figure 2 describes the detailed sample flow over the three MLSFH waves in our analysis, and Table S1 provides basic descriptive statistics for the study population used in our analyses. Mortality between waves was ascertained by the survey team when respondents could not be found for follow-up interviews during the MLSFH. Since exact dates of both deaths and transitions between ability statuses are not available, events were assumed to occur at the midpoint between survey waves. Individuals with missing information on demographic characteristics or self-reported limitation status were removed from the analysis sample.

To the best of our knowledge, the MLSFH dataset is the only dataset from a SSA LIC context that provides longitudinal health data sufficient to allow for the analyses conducted in this paper. Comparison between the 2004 Malawi Demographic and Health Survey and the 2004 MLSFH sample showed that the MLSFH was broadly representative of the overall rural population in Malawi [56], and was similar in many socioeconomic and health conditions to other LICs in SSA [42]. Comparisons of our 2010 MLSFH mature adult analysis sample characteristics to the age 45+ y rural sample of the nationally representative Malawi 2010–2011 Third Integrated Household Survey (IHS3) [57] also show that basic demographic and socioeconomic characteristics between our MLSFH study population and the IHS3 are overall quite similar (Table S1). Individuals aged 65 y and over in the MLSFH were somewhat more likely to have ever attended school than those in the IHS3, and differences arise in the distribution of religion, where Muslims are overrepresented in the MLSFH because of the fact that about one-third of the MLSFH study population is from the primarily Muslim region of Balaka.

We investigated how disabilities due to poor health result in limitations in the day-to-day activities of individuals in domains that are essential in this subsistence-agriculture context. Individuals were categorized in three different levels of functional limitations (disability states) at each MLSFH wave based on two questions from the SF-12 module about specific limitations resulting from poor physical health (with response categories being “limited a lot,” “limited a little,” or “not limited”): (1) “Please tell me if your health now limits you in carrying out moderate activities that you might do during a typical day, such as cooking and cleaning, walking to meetings in the village, or tending to cattle and livestock? If so, how much?” and (2) “Please tell me if your health now limits you in carrying out strenuous activities that you might do during a typical day, such as carrying heavy loads, working on the farm, pounding maize, or digging a pit latrine. If so, how much?”

While both groups of activities can be limited as a result of poor physical health, and the above measures were thus correlated (Spearman rank correlation = 0.56), the physical demands of these activities are not identical. The first set focuses on activities that require an ability to sustain a moderate amount of physical effort for a longer period of time, while the second set of activities requires a larger ability to exert physical strength. We constructed a three-level parameterization of functional limitations in daily living activities, which we also refer to as disability states: respondents who indicated that they had no limitations in either set of activities were classified as healthy, respondents who answered “somewhat limited” on either question were classified as moderately limited, and respondents who answered “limited a lot” on either question were classified as severely limited. In addition, 29 individuals who were assessed by interviewers as being too ill or weak to respond to the MLSFH questionnaire were coded as severely limited. As a robustness check, we tested two alternate categories of disability state in supporting information: (1) a simpler two-level measure of functional limitation (healthy versus limited) instead of the three-level characterization of disability described above, and (2) a disability measure based on the extent to which pain causes functional limitations during daily work activities.

### Statistical Analysis

We used a multi-state life table (MSLT) to translate the health transition probabilities estimated from longitudinal data to HEs. Our estimation methods are based on an adapted version of the Stochastic Population Analysis for Complex Events program [58]. Specifically, to calculate MSLT functions such as HEs, we used microsimulation [31],[59]–[63]. We created synthetic cohorts of 100,000 45-, 55-, 65-, and 75-y-old individuals with the same initial gender and functional limitation distributions as our study population (Table S4). We then “aged” these individuals forward year by year using age- and gender-specific mortality rates and probabilities of transitioning in and out of disability that were estimated from the MLSFH. This process was then repeated at each age until death. The process is essentially the microsimulation equivalent of projecting the initial synthetic cohort population **P**, disaggregated by age, sex, and health status, using **P** *_t_* = **Q·P** *_t_*, where **Q** is a projection matrix **Q** containing all age- and gender-specific health transitions rates and mortality rates [64]. After this process is applied to all individuals, the resulting synthetic cohort is analyzed to estimate HEs and other life-course health indicators. Point estimates shown are from transition probabilities and HEs estimated from the full sample. In the microsimulation approach, HE estimates are not a deterministic function of the transition rates, and instead result from the interplay between disability status, gender, and age as individuals move year by year through the simulation. Thus, the confidence intervals (CIs) from our transition rate calculations are not directly applicable to our HE estimates. CIs for HEs, which reflect both the uncertainty of the estimated parameters and the uncertainty from the microsimulation, were created by re-estimating the above analysis sequence (estimating state-dependent transition probabilities and applying them to a representative 100,000-person cohort using microsimulation) using 499 bootstrap resamples of the original dataset, and incorporating stratification by village to account for complex sample design [65]. To obtain our final 95% CIs, we took the central 95% of the distribution of these bootstrapped parameters.

We estimate the conditional probabilities of experiencing a health transition between the three disability states (healthy, moderately limited, severely limited) and death as a function of age and gender, using a logistic discrete-time hazard model of the form
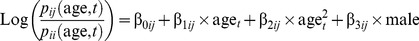
(1)where *p_ij_*(age,*t*) is the transition probability from current health state *i* (with *i* = healthy, moderately limited, severely limited, deceased) to health state *j* (with *j* = healthy, moderately limited, severely limited, deceased) over the interval from time *t*−1 to *t*, β_0*ij*_ is the intercept, β_1*ij*_ and β_2*ij*_ are the coefficients for age and age squared, and β_3*ij*_ is the coefficient for male. Transition probability estimates were obtained using PROC SURVEYLOGISTIC in SAS version 9.3 (SAS Institute), accounting for variation at region, village, and individual level.

Analyses of attrition dependent on observable characteristics found that attrition in the MLSFH was negatively related to increasing physical limitation and age. Because these variables (along with sex) are present in our analysis model, the analyses appropriately account for censoring and attrition conditional on these observable characteristics. Under the assumption of conditional ignorability, differential attrition related to these included characteristics does not distort our findings [66]. Multiple imputation of missing data by chained equations [67],[68] was conducted to evaluate whether the analyses were robust with respect to alternative assumptions regarding missingness of the data.

## Results

### Observed Health and Work Effort

Table 1 reports summary statistics of the analysis sample and the distribution of self-reported disability status across the three MLSFH survey rounds during 2006–2010. With the introduction of the parent sample in the 2008 MLSFH, the mean age increased by 5 y, and the sex composition shifted from being majority male to majority female. As the sample population aged, the incidence of functional status limitations rose—by wave 6, the majority of respondents had some limitations on their activities. For example, in 2010, close to one-third of respondents aged 45–64 y indicated that they were moderately limited, and 8.5% severely limited, in their physical activities, with both physical limitation states being substantially more common among individuals aged 65+ y (Table 1). Although the measures of physical limitation are not directly comparable, this basic pattern of high levels of disability among mature adults that increase rapidly with age is also found in the IHS3 (Table S1).

**Table 1:** Self-reported disability, percentage working for income in the past 2 weeks, pain interfering with work, and subjective well-being for mature adults (aged 45+ y) in the MLSFH.

Age Group	Characteristic	Year						Percent (2010) Who Worked for Income in Past 2 Weeks		Percent (2010) for Whom Pain Interfered with Work in Last 4 Weeks		Percent (2010) Who Are Somewhat/Very Unsatisfied with Life	
		2006		2008		2010							
		*N*	Percent	*N*	Percent	*N*	Percent	*N*	Percent	*N*	Percent	*N*	Percent
**45–64 y**	**Disability Status**												
	Active	427	72.4%	537	67.4%	428	56.8%	165	38.5%	64	14.9%	16	3.7%
	Moderately limited	132	22.4%	200	25.1%	239	31.7%	82	34.3%	120	50.2%	23	9.6%
	Severely limited	31	5.3%	41	5.1%	64	8.5%	16	26.2%	40	65.6%	16	26.2%
	Dead	—	—	19	2.4%	23	3.1%	—	—		—		—
	**Men**	316	53.6%	362	45.4%	340	45.1%	—	—		—		—
**65+ y**	**Disability Status**												
	Active	34	54.0%	120	41.0%	87	24.6%	23	26.4%	18	20.6%	5	5.7%
	Moderately limited	20	31.7%	108	36.9%	142	40.1%	34	23.9%	75	52.8%	14	9.9%
	Severely limited	9	14.3%	62	21.2%	93	26.3%	8	9.0%	69	77.5%	26	29.2%
	Dead	—	—	3	1.0%	32	9.0%	—	—		—		—
	**Men**	43	68.3%	142	48.5%	170	48.0%	—	—		—		—
**Average Age**		54.2		59.0		61.0							

Disability classification is based on MLSFH questions (1) “Do you have any health problems that limit you in carrying out moderate activities?” and (2) “Do you have any health problems that limit you in carrying out strenuous activities?”, with each question providing a list of moderate/strenuous activities and response categories being “not limited,” “limited a little,” and “limited a lot.” Individuals who indicated that they had no limitations in either set of activities were classified as healthy, those who responded “somewhat limited” on either question were classified as moderately disabled, and individuals who responded “limited a lot” on either question were classified as severely disabled. “Dead” refers to mortality between survey waves among respondents who were interviewed in the MLSFH 2006 and/or 2008. “Pain interfering with work” is based on the question “During the past 4 wk, how much did pain interfere with your normal work (including both work outside the home and housework)?” Individuals responding “moderately,” “quite a bit,” or “extremely” were classified as being limited by pain. Individuals responding “somewhat unsatisfied” or “very unsatisfied” to the question “How satisfied are you with your life, all things considered?” were classified as having low life satisfaction. Three severely limited individuals aged 45–64 y and four severely limited individuals aged 65+ y had missing values on working for income, pain interfering with work, and life satisfaction, and were removed from the denominator for percentage calculations.

Using data on time use, analyses of the MLSFH indicate that functional limitations and disabilities are associated with substantially reduced work efforts. For example, the percentage of individuals working for income within the past week decreased steadily with increasing disability (Table 1): individuals aged 45–64 y were over 12 percentage points less likely (26% versus 39%) to have engaged in work for income in the past week if they were severely limited, and severely limited individuals 65 y and over were over 17 percentage points (9% versus 26%) less likely to work for income. Individuals who reported limitations on physical activity also reported that their work efforts (both within and outside the household) had been substantially limited by pain, with around 50% of moderately and 60%–80% of severely limited individuals stating that pain interfered quite a bit or extremely with their normal work during the past 4 wk (Table 1). These gradients in economic activity by disability status persisted in regression analyses that controlled for age and gender, and additional regression analyses showed that increasing disability was also associated with fewer hours of family farm work, for example, controlling for age and gender, severely limited individuals contributed an average of 5.32 fewer hours of agricultural labor to the household per week (Table S2). For a 55-y-old man, this represents nearly a halving of farm labor contribution compared to a healthy man.

Physical limitation is also associated with substantially lower subjective well-being, with more than a quarter of severely limited individuals responding that they were “somewhat unsatisfied” or “very unsatisfied” with their lives, as compared to less than 4% of healthy individuals 45–64 y and less than 6% of healthy individuals 65+ y (Table 1), a pattern that persists after controlling for age and gender (Table S2).

### Health Transition Probabilities

**Figure 3:**
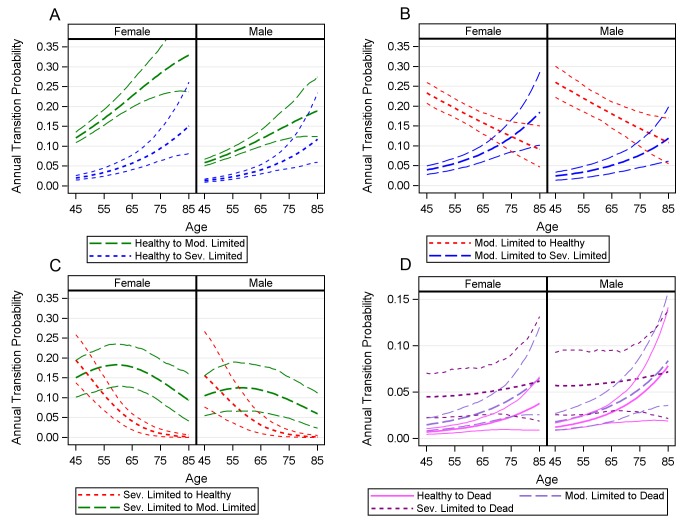
Estimated annual transition probabilities. (A) The annual probability of transitioning from healthy to moderately limited (mod. limited) and severely limited (sev. limited). (B) The annual probability of transitioning from moderately limited to healthy and severely limited. (C) The annual probability of transitioning from severely limited to healthy and moderately limited. (D) Annual probability of mortality for healthy, moderately limited and severely limited individuals.

Analysis of the 2006–2010 MLSFH reveals that individuals in this population experienced a relatively large number of transitions between different disability states (healthy, moderately limited, severely limited) and death during the period of observation. Among respondents observed for all three MLSFH waves, 59% experienced at least one and 22% experienced two transitions between different disability states. Table S3 shows the distribution of all transitions between disability states that were observed in our study population during the period 2006–2010. To gain insight into the dynamics of disability in this population, we modeled the underlying age- and gender-specific annual transition probabilities between states of physical limitation. Figure 3A illustrates the annual transition probabilities out of healthy status by sex, along with the 95% CIs based on 499 bootstrap resamples. At older ages, particularly above age 75 y, CIs around the point estimates are quite large, primarily as a result of limited sample sizes at these advanced ages. As expected, transition probabilities towards increased physical disability rise sharply with age—a healthy man at age 45 y has less than a 0.08 probability of being anything but healthy at age 46 y, but a healthy man at age 75 y has only a 0.73 chance of remaining healthy at age 76 y. These figures are sharply higher for women, following a widely established pattern in other populations where disability and functional limitation transitions have been studied [69],[70]. From healthy status, women are significantly more likely to enter into moderately limited status at all ages than are men, and are also significantly more likely to become severely disabled until about age 75 y.

The moderate limitation state is characterized by relatively high rates of entry and exit, though the probability of the direction of these transitions (upwards to severe limitation, downwards to healthy) changes substantially with age. Figure 3B describes the annual transition probabilities for a moderately limited individual at ages 45–85 y, along with the 95% CIs around these estimated probabilities. At younger ages, individuals of both sexes are relatively likely to recover from stays in moderate limitation. The probabilities of recovery decline sharply with age, however: by about 70 y, women are more likely to become severely limited or die than to recover to healthy life (the corresponding age for men is about 78 y). Substantial sex differences in transition probabilities arise here as well—women are significantly more likely to become severely disabled and significantly less likely to remain healthy at almost all ages.

Transition probabilities from the severely limited state (Figure 3C) provide evidence that this is a fairly retentive state and that with increasing age, probabilities of exiting severe limitation by transitioning to any state other than death decline sharply. As would be expected given the severity of the state, the probability of experiencing a full recovery from this state declines sharply over time: after age 49 y for women (52 y for men), recovering individuals are more likely to move to the moderately limited state than to fully healthy, and after age 64 y for women (59 y for men), individuals are more likely to die than to recover to healthy status. For both men and women, the probability of full recovery to healthy life is almost zero after age 75 y. In the SSA LIC context studied here, functional limitations have a strong association with mortality (Figure 3D). At younger ages in particular, annual mortality rates among individuals with severe limitation are orders of magnitude higher than for those in healthy or moderately limited states—a 45-y-old severely limited woman is about six times more likely to die than a healthy woman (5.3 times for a man). Point estimates for transitions to mortality from healthy status are higher for men than for women at all ages, although the 95% CIs overlap. Overall, severe disability seems more predictive of death in men than in women, though given the small number of observed deaths, the CIs around these estimates are fairly large.

As a robustness check to verify that the estimated transition probabilities based on the MLSFH (Figure 3) reflect longer term trends in health transitions in the population, we compared the age-specific proportions in each health status in both the MLSFH sample and the synthetic cohorts created through microsimulation (Figure S2). We found that the simulated data, based on the predicted parameters estimated from the MLSFH sample, matched quite closely with actual observed proportions in the sample population. The proportions from the simulated data lie entirely within a 95% CI around the MLSFH sample proportions (estimated through 999 bootstrap samples) except for the proportion of healthy 75+-y-old women, for which the microsimulation data predict fewer healthy women than is observed in the data. However, the higher proportion of healthy individuals observed in the sample population is likely a result of the small sample of individuals in this age group. The parametrically based microsimulation cohort likely more accurately represents the conditions in this rural population.

### Health Expectancies

**Figure 4:**
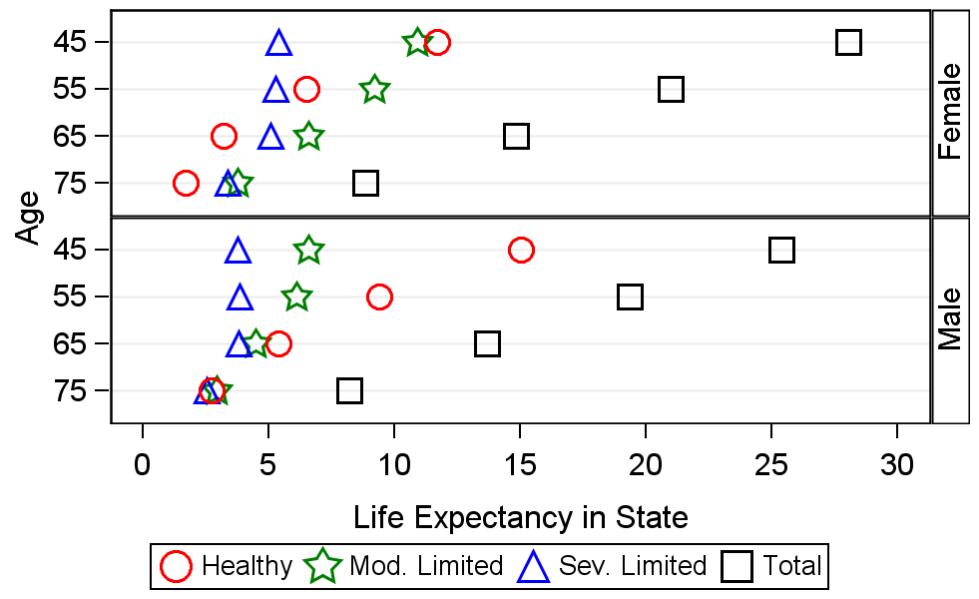
Average number of years of active, moderately limited, and severely limited life expectancy, and total life expectancy. This figure shows a comparison between the number of years an average individual will spend in healthy, moderately limited (mod. limited), and severely limited (sev. limited) life at age 45, 55, 65, and 75 y. Markers represent the overall distribution of life-years spent in each state, not the ordering of these life-years; individuals in our analysis can recover and relapse between disability states, so not all years of limitation are spent at the end of life.

**Figure 5:**
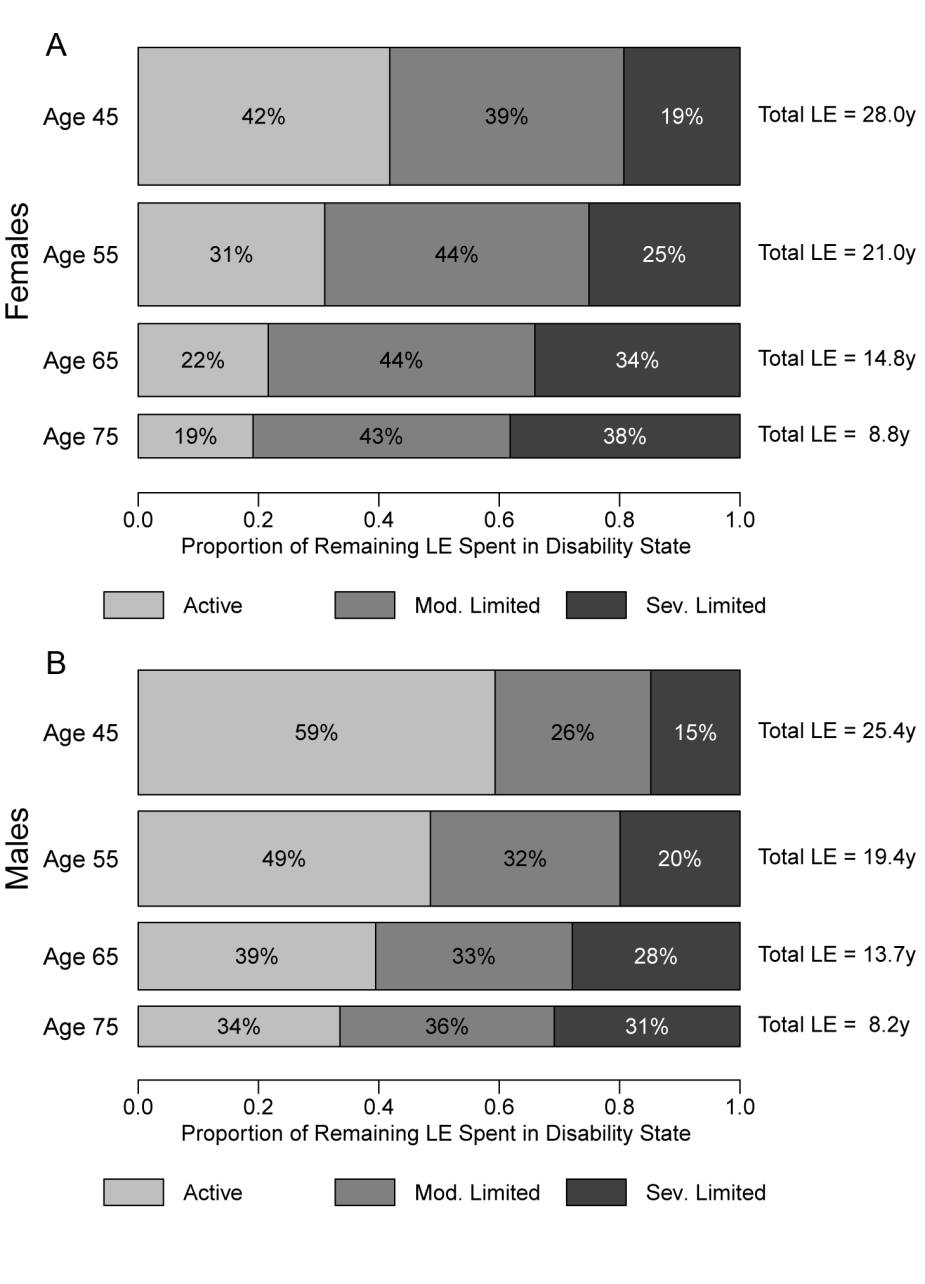
Distribution of remaining life expectancy by disability state: healthy, moderately limited, and severely limited. This figure shows the proportion of remaining life an average individual will spend in healthy, moderately limited (mod. limited), and severely limited (sev. limited) life at age 45, 55, 65, and 75 y, for women (A) and men (B). The height and area of each bar is proportional to the overall remaining LE of the synthetic cohorts with initial ages of 45, 55, 65 and 75 y, and the differently shaded areas represent the distribution of the remaining LE across the three disability states: healthy, moderately limited, and severely limited. The bars do not necessarily reflect the ordering of these life-years by disability states, as individuals in our analysis can recover and relapse between disability states, so not all years of limitation are spent at the end of life.

Moving from the transition probabilities in Figure 3 to the corresponding HEs (Table 2; Figures 4 and 5) translates the age- and sex-specific annual probabilities of transition between disability states into years lived with functional limitation. As a validation test for our analyses, we compared the total LE figures to 2009 World Health Organization [71] and 2008 Malawi National Census [72] life tables (Figure S3). The microsimulation-based MLSFH LE estimates are in broad alignment with these other data sources, especially in light of the fact virtually all LE estimates at higher ages in SSA LIC contexts are subject to considerable uncertainty due to the lack of reliable vital registration data.

**Table 2:** Microsimulation-estimated average remaining life expectancy at ages 45–75 y, by sex.

Age	Life Expectancy by Sex	Estimate	95% CI
**45 y**	**Women**		
	Total	28.04	(25.71–33.49)
	Active	11.72	(10.43–13.77)
	Mod. limited	10.90	(9.44–13.39)
	Sev. limited	5.41	(4.31–7.06)
	**Men**		
	Total	25.39	(23.31–28.83)
	Active	15.06	(13.61–16.92)
	Mod. limited	6.57	(5.39–8.49)
	Sev. limited	3.76	(2.78–5.36)
**55 y**	**Women**		
	Total	20.97	(19.19–31.72)
	Active	6.50	(5.44–9.50)
	Mod. limited	9.21	(8.04–14.48)
	Sev. limited	5.26	(4.23–7.79)
	**Men**		
	Total	19.38	(17.51–25.00)
	Active	9.41	(8.19–11.80)
	Mod. limited	6.11	(4.98–8.45)
	Sev. limited	3.86	(2.81–5.46)
**65 y**	**Women**		
	Total	14.85	(13.46–21.61)
	Active	3.21	(2.45–4.34)
	Mod. limited	6.58	(5.49–9.84)
	Sev. limited	5.06	(3.98–6.92)
	**Men**		
	Total	13.70	(12.37–16.08)
	Active	5.41	(4.42–6.72)
	Mod. limited	4.48	(3.60–6.19)
	Sev. limited	3.82	(2.76–5.40)
**75 y**	**Women**		
	Total	8.85	(8.20–15.14)
	Active	1.69	(1.05–2.99)
	Mod. limited	3.78	(2.89–7.24)
	Sev. limited	3.38	(2.54–5.19)
	**Men**		
	Total	8.23	(7.54–12.73)
	Active	2.76	(1.96–4.10)
	Mod. limited	2.93	(2.27–5.37)
	Sev. limited	2.54	(1.83–3.72)

Estimates were obtained from synthetic cohorts of 100,000 45-, 55-, 65-, and 75-y-olds created via microsimulation, based on observed transition rates from 2006–2010 MLSFH data.

mod. limited, moderately limited; sev. limited, severely limited.

In addition to revealing remaining total LE for mature adults, our microsimulation-based MSLT approach estimates the duration of life expected to be spent in healthy, moderately limited, and severely limited statuses. To our knowledge, information about HEs in different disability states was not available for Malawi or similar SSA LIC contexts prior to this study. Specifically, the estimated HEs in Table 2 and Figures 4 and 5 show that that mature adults in rural Malawi are expected to live a substantial number of their remaining life years—and thus a significant fraction of their remaining LE—subject to functional limitations and in a state of moderate or severe disability. For example, our analyses show that the average 45-y-old woman is expected to live about 28 additional years, making her expected age at death almost 73 y (the corresponding LE estimates for the average 45-y-old man are 25.4 additional years and an expected age at death of 70.4 y). However, almost 60% of the 28 y for the average woman will be lived with some limited functional status, while the average man will be limited for about 40% of his remaining life. The high rates of transition between health states across the life course mean that time spent in limited status does not occur solely at the end of life. Analysis of HEs in the simulated synthetic cohort of 45-y-olds shows that, on average, a woman at age 45 y will spend 2.7 y in moderate limitation and 0.6 y in severe limitation before she reaches age 55 y (these corresponding values for men are 1.6 and 0.4 y). Figure 4 emphasizes the age trends in years of LE in healthy, moderately limited, and severely limited life. By 65 y, women can expect to live only 3.21 y of their remaining 14.85 y of life without functional limitation, and men can expect to live only 5.4 remaining years without functional limitations. Figure 5 displays the proportions of remaining LE spent in each disability state by age, showing a clear and progressive increase in the amount of remaining life spent moderately or severely limited. By 75 y, women are expected to live over 80% of their remaining life in some limited condition (this value is 66% for men).

Calculating HEs from only the portion of the synthetic cohort starting in a given health state, as compared to the full empirical distribution of disability states observed at the various ages in the MLSFH (Table S1), has relatively small effects on the overall LE and the fraction of the remaining life spent in the various disability states for cohorts starting at age 45 y or age 55 y (Figures S4, S5, S6). At older starting ages, starting with an initial healthy state increases LE and reduces the proportion of the remaining life with disability. Starting with moderate or severe limitations, as is expected, reduces remaining LE and increases the fraction of remaining LE lived with disability.

To evaluate whether our results in Table 2 and Figures 4 and 5 are affected by attrition in the longitudinal MLSFH study, the above analyses were replicated using multiple imputation by chained equations [67],[68] to impute missing follow-up data (Table S5). The results using imputed values (Table S5) are in close agreement with the above results that were obtained without multiple imputation (Table 2; Figures 4 and 5). The primary difference is that the multiple imputation models estimate a slightly shorter healthy LE for men at age 45 and 55 y, and a slightly shorter total LE for both sexes at age 45 y. None of these differences affects the substantive conclusions obtained from our analyses. Our conclusions are also robust with respect to using a two-state rather than three-state classification of functional limitations and disability, and our overall conclusions do not change when using a classification of disability based on functional limitations resulting from pain during daily work activities (Tables S6 and S7).

## Discussion

Older individuals have received inadequate attention in much of the current health-related research in low-income sub-Saharan contexts, despite the fact that poor health in this population is common, levels of disability are high, and economic productivity is often hindered because of persistent health-related functional limitations. While national health sector strategic plans in Malawi and other SSA countries have started to highlight the need for policies to prevent disabilities and ensure access to curative and rehabilitative care among older individuals, there is only a limited understanding of the trajectories of health and disability among mature and elderly adults in SSA, and of the health needs that will result from the oncoming growth of the mature adult and elderly population in many SSA contexts. The required health sector responses to population aging in SSA are thus inadequately informed by the existing literature, which has often focused on the health of younger individuals and/or health concerns resulting from infectious diseases, rather than chronic and/or noncommunicable diseases and disabilities that affect older individuals.

The key contribution of this paper is its focus on the lived experience with disability among mature adults in rural Malawi, including both the levels of disability by age and the dynamics of disability transitions during the adult life course. Our analyses do not single out HIV-infected individuals. Rather, we treat this disease as one of many health concerns—chronic diseases, accidents, physiological aging, etc.—that affect mature adults in SSA. Specifically, we investigate how overall physical health results in functional limitations, that is, how disabilities due to poor health result in limitations in day-to-day activities in domains that are essential for individuals in this subsistence-agriculture context. Individuals are categorized into three different disability states based on the functional limitations that they experience. Using a novel MSLT methodology, which has not (to our knowledge) been applied to this context before, this study provides insight into the processes of functional limitation in a rural SSA population by estimating the prevalence of functional limitations and the transition rates between different disability states. Our analyses find that levels of disability and functional limitations in this population are very high, and that rates of transition into disability statuses differ substantially across the life course. Rates of recovery from moderate and severe limitations decline very rapidly with age, and after age 65 y a full recovery from severe functional limitations is very unlikely.

In addition to documenting the levels of disability and the transitions between disability states, our analyses estimate the expected years people in this rural population will live in healthy, moderately limited, and severely limited life. For example, we estimate that women at age 45 y will spend 58% of their remaining life with moderate or severe functional limitations, a fraction that rises to 78% at age 65 y; 45-y-old men are expected to spend 41% of their remaining life with moderate or severe functional limitations, rising to 60% at age 65 y. Our measures of functional limitation are chosen to be appropriate for the physically demanding environment of a subsistence-agriculture lifestyle, making comparisons with estimated levels of disability in developed regions somewhat difficult. Even so, such comparisons may be useful if we conceptualize disability as the inability to fully physically function in one's environment. The proportions of remaining life expected to be spent with severe limitations at age 45 y in Malawi are comparable to those of 80-y-olds in the US, and the proportions of life with any limitations are far higher [73],[74]. Time spent in physical limitation is widely distributed across ages in our study population, with a substantial number of expected years of limitation occurring before age 55 y. Although our dataset did not include specific data on chronic disease, it is likely that chronic diseases combined with lifelong exposure to multiple infectious diseases, frequent poverty, and widespread poor nutrition contribute to the disabilities observed [75]–[77].

The microdata-based analyses in this article represent a substantial methodological shift from previous measures of healthy LE calculated for SSA, in particular those of the Global Burden of Disease reports [41]. Though the Global Burden of Disease estimates of healthy LE provide a useful metric for cross-national comparisons, the metric of healthy LE at birth is not readily applicable to the life-course experience of an adult. The methodology used in this study allows for deeper insight into processes of disability than is provided by the Global Burden of Disease, more accurately characterizing the fluidity between health states over time and across the population. These results show that individuals in this society experience a lengthy struggle with disabling conditions in later life, with high probabilities of remitting and relapsing between states of limitation. This level of in-depth understanding of this population's burden of disease is possible only through analysis of microdata.

In evaluating the results from this MSLT estimation, which to our best knowledge has been applied in this study for the first time to analyzing health transitions in SSA, several limitations need to be considered. Individuals who experience a health transition between MLSFH waves are assumed to experience only a single transition during the 2-y period between surveys, which likely misses shorter term transitions between health statuses. As the focus of this article is on functional limitations (which tend to be longer in duration) and not acute health conditions, we are reasonably confident that this assumption does not unduly bias our HE estimates and their interpretation [78],[79]. The MLSFH does not provide data on the individual diseases or medical conditions that result in functional limitations, limiting our ability to identify specific causes of the high disability burden we observe. In common with other life-table-based measures, HE estimates assume stationary transition rates over time—that is, they apply to the lived experience of a synthetic cohort in which the estimated age-specific rates of transition remain constant for the foreseeable future—and thus will not exactly match the lived experience of any single cohort. Thus, our results do not take into account any shifts in disability prevalence that may have occurred during the 4-y study period. Our current analyses follow a first-order Markov chain, and are thus not state-duration-dependent—that is, transition probabilities are not adjusted by duration of stay in a given state. This assumption results from the left-censored nature of our data—though we can determine what functional status individuals had at entry into the dataset, we do not know their duration of stay in that state. Recent work on the semi-Markov process expectation maximization algorithm [80] rectifies some of the left-truncation biases introduced by state-duration-dependent modeling, but was deemed too computationally intensive and complex given the sample size available in the MLSFH.

Our findings suggest that the high burden of functional limitations and disabilities experienced by this rural mature adult population results in a substantial gap between potential and actual economic productivity. Functional limitations are associated with a lower likelihood of working for income and reduced work efforts in agriculture, key aspects of individuals' livelihoods in rural SSA LIC contexts. Given the lack of institutionalized social and economic support systems, Malawi and other SSA LICs can ill afford this productivity gap resulting from functional limitations among mature adults at already “relatively young old ages.” The significant productivity role of mature adults in rural Malawi was summarized at the inauguration of the Malawi Ministry of Persons with Disabilities and the Elderly: “[M]any older people are able to make significant contributions as income-earners, providers of care, sources of knowledge and experience, and guardians of traditions. Since the effects of the ageing process are certain to continue for many years to come, agriculture and rural development will be increasingly dependent on older persons. Therefore, policy makers must find better ways to ensure that older people are able to ‘age successfully’: have good health, be physically and mentally active, and remain actively involved in community life” [33].

Our findings make an important contribution to the debate about policy responses and interventions targeting chronic disease and disability in low-income settings, particularly in SSA. We show that moderate and severe functional limitations—which have a substantial negative effect on individuals' economic activities—are a major challenge in the subsistence-agriculture setting that is characteristic of many rural SSA LIC contexts. However, the older population has largely been left out of the recent large-scale health-focused interventions and policies implemented in SSA, particularly those focusing on Millennium Development Goal-related groups [81]. Many policy makers in SSA are hesitant to direct financial resources to the elderly population, and see investment in health resources for the aging population as “irrelevant to core national development interests” [23]. Our analyses suggest that this sentiment is misguided—the high burden of disability among mature adults is associated with substantial loss of direct labor output, with potentially important intergenerational consequences for children and younger adult family members. Mature adults with functional limitations and related disabilities are likely to be a drain on the scarce time and material resources of families, and may contribute less time and money towards children and younger adults [27]. Investments in improving the health of this growing population have the potential to significantly improve aggregate economic growth, and our analyses provide important empirical support to recent editorial and policy papers that have argued for greater attention to mature and older adults within national health policies and international donor-funded health programs in SSA LICs [12],[14],[17],[22],[23].

## Supporting Information

Figure S1 **MLSFH study locations in Malawi.**
(TIF)

Figure S2 **Proportions in healthy, moderately limited, and severely limited states in MLSFH data and microsimulation cohort, by sex.** The figure shows a comparison between the age-specific proportions in each health state in both the MLSFH sample (solid lines) and the synthetic cohorts created through microsimulation (dashed lines). To smooth these data and gain an insight into the overall patterns of disability, we used a local non-parametric linear regression procedure (PROC LOESS) in SAS version 9.3.
(TIFF)

Figure S3 **Remaining life expectancy in various life tables, Malawi (men and women).** MLSFH LEs are estimated based on the 2006–2010 MLSFH mature adult population using the microsimulation-based Stochastic Population Analysis for Complex Events MSLT approach described in the main text. World Health Organization LE estimates for Malawi are estimated using model life tables that are calibrated using infant/child mortality levels [71]. 2008 Malawi census LE estimates are obtained from a life table combining estimates of infant and child mortality with age-specific mortality rates derived from household death data, adjusted for underreporting of deaths [72].
(TIF)

Figure S4 **Average number of years of active, moderately limited, and severely limited life, and total life expectancy, conditional on initial disability status.** The graphs in this figure show a comparison between the number of years an average individual will spend in healthy, moderately limited, and severely limited life at age 45, 55, 65, and 75 y, conditional on initial disability status (i.e., disability status at age 45, 55, 65, or 75 y for the portion of the simulated synthetic cohort is initially healthy [A], moderately limited [B], or severely limited [C]). As in Figure 4, the markers represent the overall distribution of remaining life-years spent in each state, not the ordering of these life-years; individuals in our analysis can recover and relapse between disability states, so not all years of limitation are spent at the end of life. Differences in overall LE between (A), which conditions on all individuals being healthy at age 45 y, and Figure 4, in which members of the synthetic cohort have the full empirically observed distribution of disability states as shown in Table S4, are relatively small for cohorts beginning at age 45 or 55 y. This is due to two facts: first, at these ages a large fraction of the MLSFH population, and thus the initial health states of the simulated cohorts in Figure 4, is in the healthy state (see Table S4), and, second, at these relatively young ages, the probability of recovering from a moderately limited or severely limited state is relatively high (Figure 3). Therefore, an initial moderately—and with lower probability also a severely—limited state is likely to be transient. At older ages (i.e., a starting age of 65 or 75 y for the synthetic cohort), the differences between Figure 4 and (A) increase as more individuals in Figure 4 enter the synthetic cohort with moderate or severe limitations, and these disabilities become increasingly persistent at older ages (Table S4; Figure 3). Hence, conditioning on a healthy initial state in the synthetic cohorts (A) has the result that individuals live longer and live a larger fraction of their remaining life in the healthy or moderately limited state. Independent of the initial age (45, 55, 65, or 75 y) of the synthetic cohort, conditioning on an initial moderate or severe limitation (B and C) has the result that the overall LE of individuals in the synthetic cohort declines, and that individuals spend a larger fraction of their remaining lives (see Figures S5 and S6) with moderately or severe functional limitations, as compared to the synthetic cohort with the full observed distribution of disability states (as in Figure 4) or with individuals initially only in the healthy state (as in [A]). This reduction in LE, and the shift towards spending a larger fraction of the remaining life with disability as compared to (A) and Figure 4, is most pronounced in (C), where all members begin in a severely limited state.
(TIF)

Figure S5 **Women: distribution of remaining life expectancy by disability state (healthy, moderately limited, severely limited), conditional on initial disability status.** This figure shows the proportion of remaining life an average individual will spend in healthy, moderately limited, and severely limited life at age 45, 55, 65, and 75 y, conditional on the initial disability status for individuals in the synthetic cohort being healthy (A), moderately limited (B), or severely limited (C). The height and area of each bar is proportional to the overall remaining LE of the synthetic cohorts with initial ages of 45, 55, 65, and 75 y, and the differently shaded areas represent the distribution of the remaining LE across the three disability states: healthy, moderately limited, and severely limited. The bars do not necessarily reflect the ordering of these life-years by disability states as individuals in our analysis can recover and relapse between disability states, so not all years of limitation are spent at the end of life.
(TIF)

Figure S6 **Men: distribution of remaining life expectancy by disability state (healthy, moderately limited, severely limited), conditional on initial disability status.** As in Figure S5, but for men.
(TIF)

Table S1 **Descriptive statistics for the 2010 MLSFH study population, and comparison of the MLSFH and the IHS3 (rural) sample characteristics.**
(PDF)

Table S2 **Regression analyses for associations between disability states and income earned, pain interference with work, dissatisfaction with life, and hours worked on farm.**
(PDF)

Table S3 **Distribution of observed transitions between disability states (healthy, moderately limited, severely limited) during 2006–2010 (three MLSFH waves).**
(PDF)

Table S4 **Initial gender and functional status distribution for the synthetic cohorts (** ***n*** **= 100,000) used in the microsimulation, as based on the MLSFH.**
(PDF)

Table S5 **Microsimulation-estimated average remaining life expectancy at ages 45–75 y, by sex, with missing data imputed using multiple imputation by chained equations** [**67**],[**68**] **.**
(PDF)

Table S6 **Microsimulation-estimated average remaining life expectancy at ages 45–75 y, by sex, using a two-level classification of disability (healthy versus limited).**
(PDF)

Table S7 **Microsimulation-estimated average remaining life expectancy at ages 45–75 y, by sex, using a classification of disability based on pain interfering with daily work activities.**
(PDF)

Table S8 **MLSFH study population 1998–2010.**
(PDF)

Text S1 **MLSFH sampling methods and related relevant data collection procedures.**
(DOC)

## Abbreviations

CI: confidence interval
HE: health expectancy
IHS3: Third Integrated Household Survey
LE: life expectancy
LIC: low-income country
MLSFH: Malawi Longitudinal Survey of Families and Health
MSLT: multi-state life table
SSA: sub-Saharan Africa
