# Supplementary figures and images for: Disability Transitions and Health Expectancies among Adults 45 Years and Older in Malawi: A Cohort-Based Model
Source: PLoS Med. 2013 May 7;10(5):e1001435. doi: 10.1371/journal.pmed.1001435 (PMC3646719; doi:10.1371/journal.pmed.1001435)

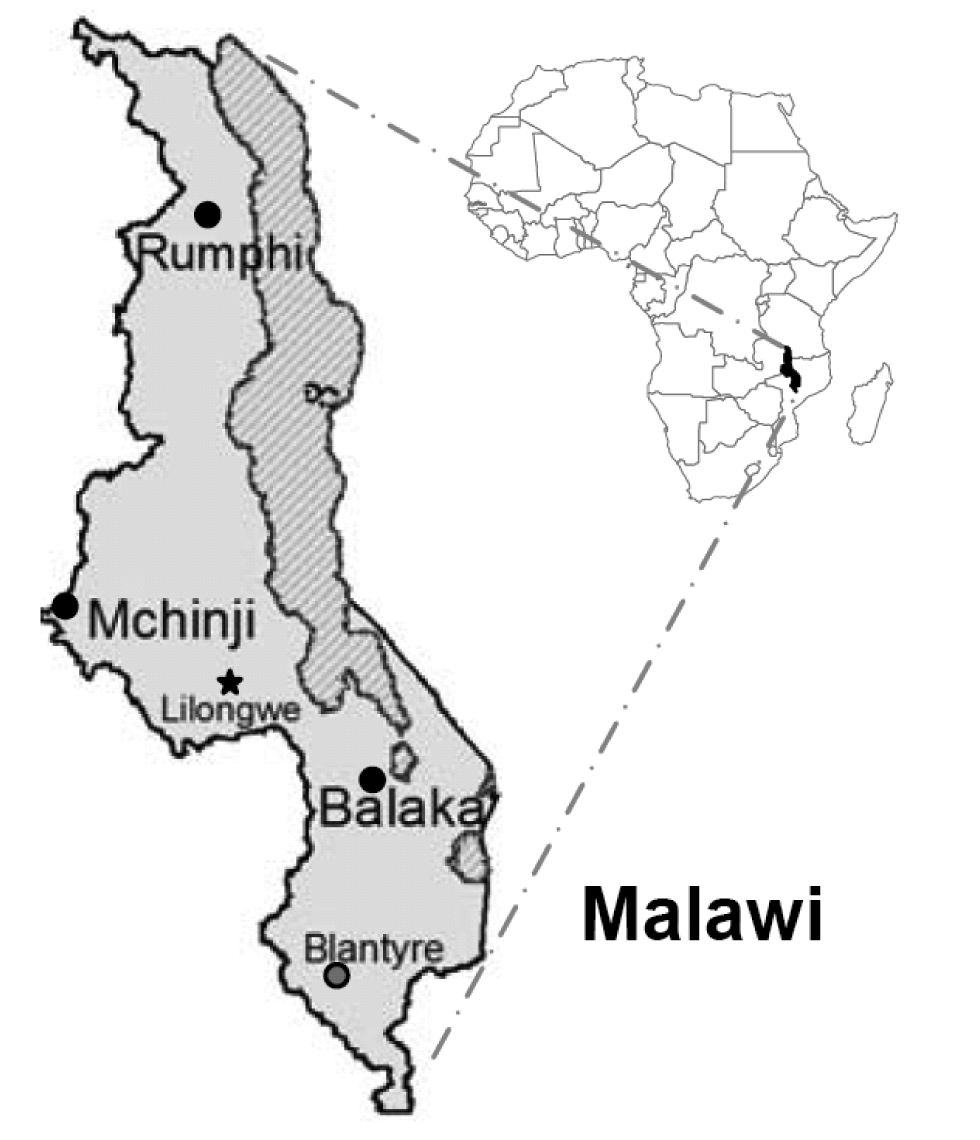

Supplement: Figure S1 — MLSFH study locations in Malawi. (TIF) [file pmed.1001435.s001.tif]

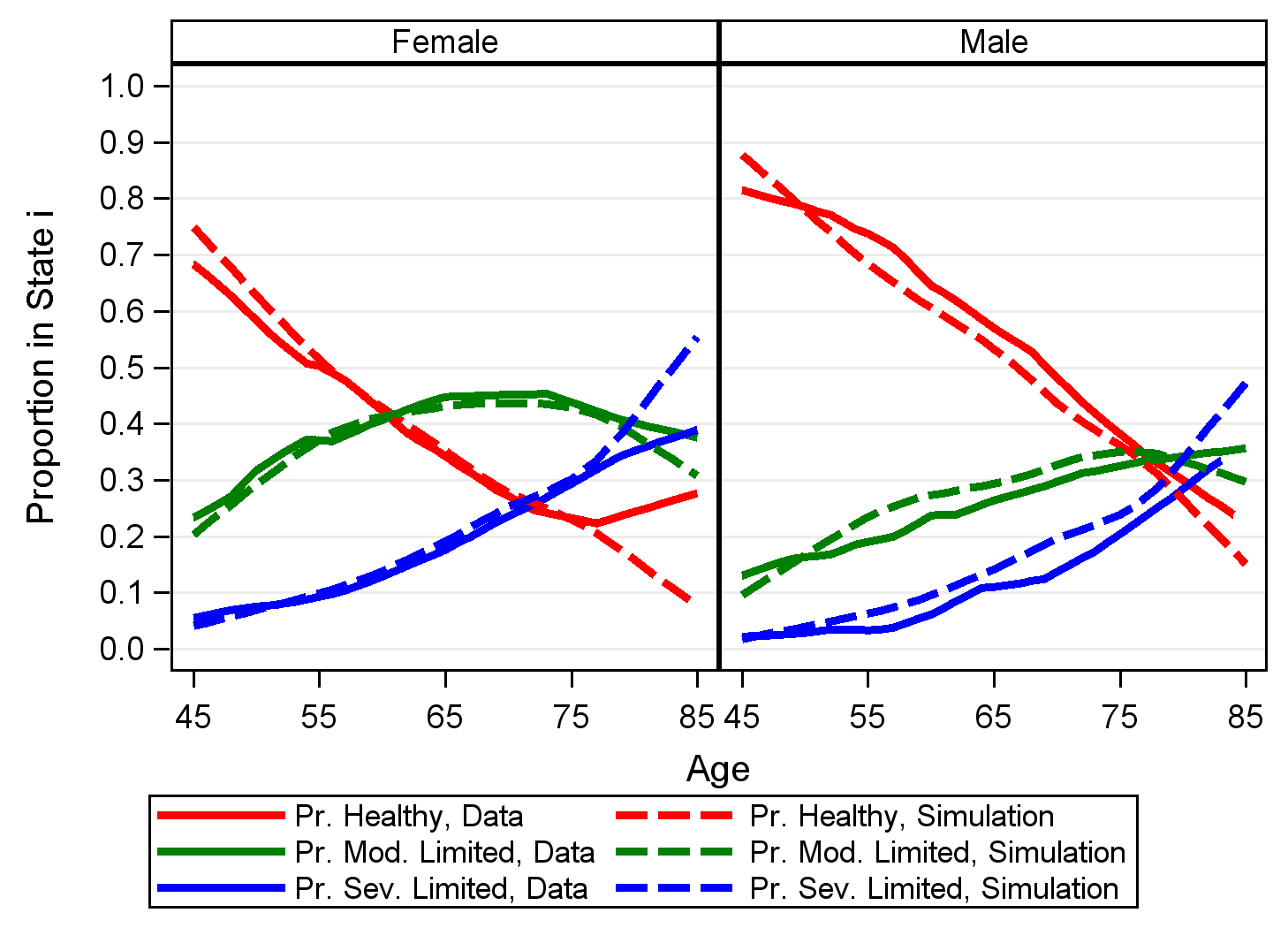

Supplement: Figure S2 — Proportions in healthy, moderately limited, and severely limited states in MLSFH data and microsimulation cohort, by sex. The figure shows a comparison between the age-specific proportions in each health state in both the MLSFH sample (solid lines) and the synthetic cohorts created through microsimulation (dashed lines). To smooth these data and gain an insight into the overall patterns of disability, we used a local non-parametric linear regression procedure (PROC LOESS) in SAS version 9.3. (TIFF) [file pmed.1001435.s002.tiff]

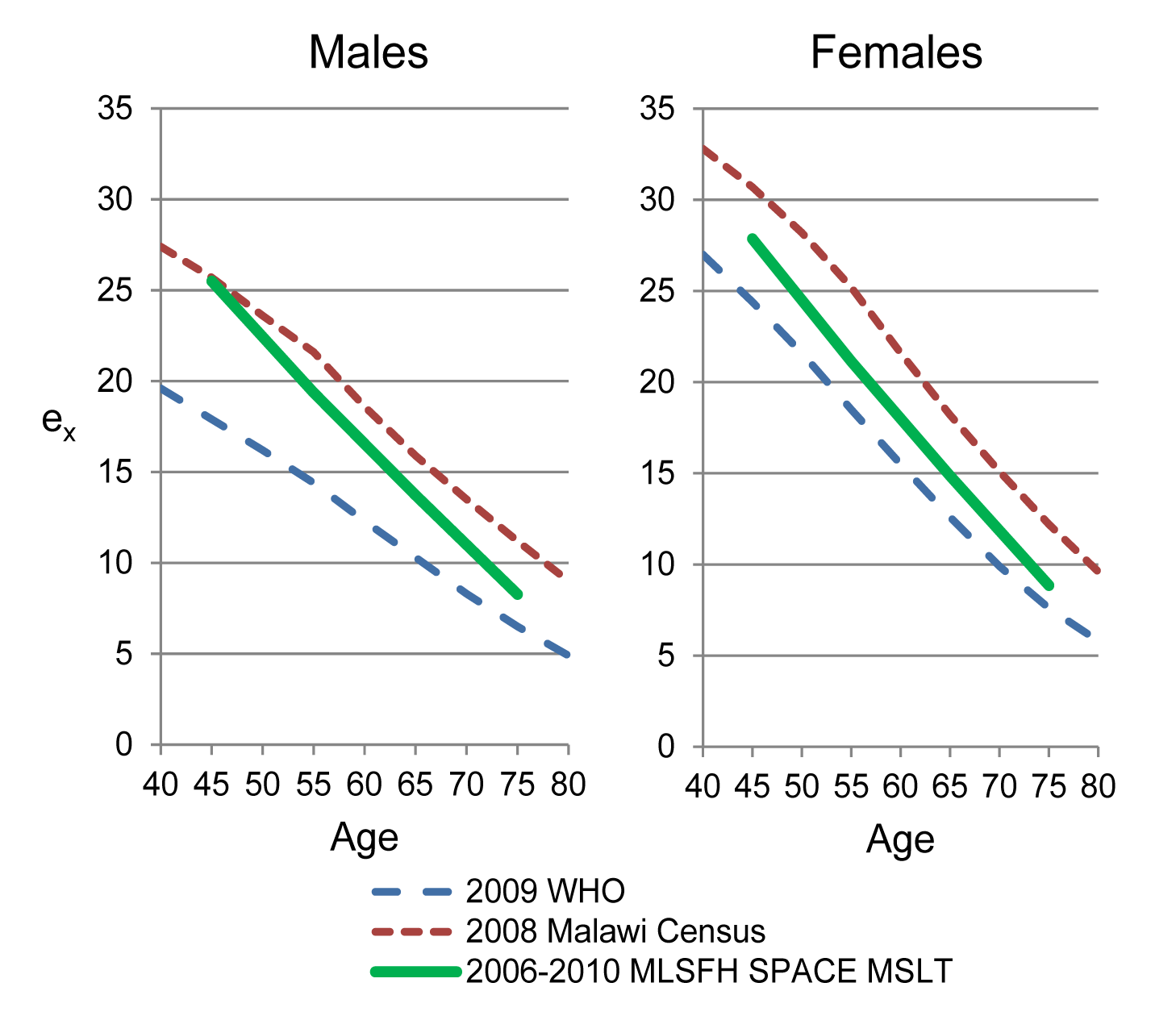

Supplement: Figure S3 — Remaining life expectancy in various life tables, Malawi (men and women). MLSFH LEs are estimated based on the 2006–2010 MLSFH mature adult population using the microsimulation-based Stochastic Population Analysis for Complex Events MSLT approach described in the main text. World Health Organization LE estimates for Malawi are estimated using model life tables that are calibrated using infant/child mortality levels [71]. 2008 Malawi census LE estimates are obtained from a life table combining estimates of infant and child mortality with age-specific mortality rates derived from household death data, adjusted for underreporting of deaths [72]. (TIF) [file pmed.1001435.s003.tif]

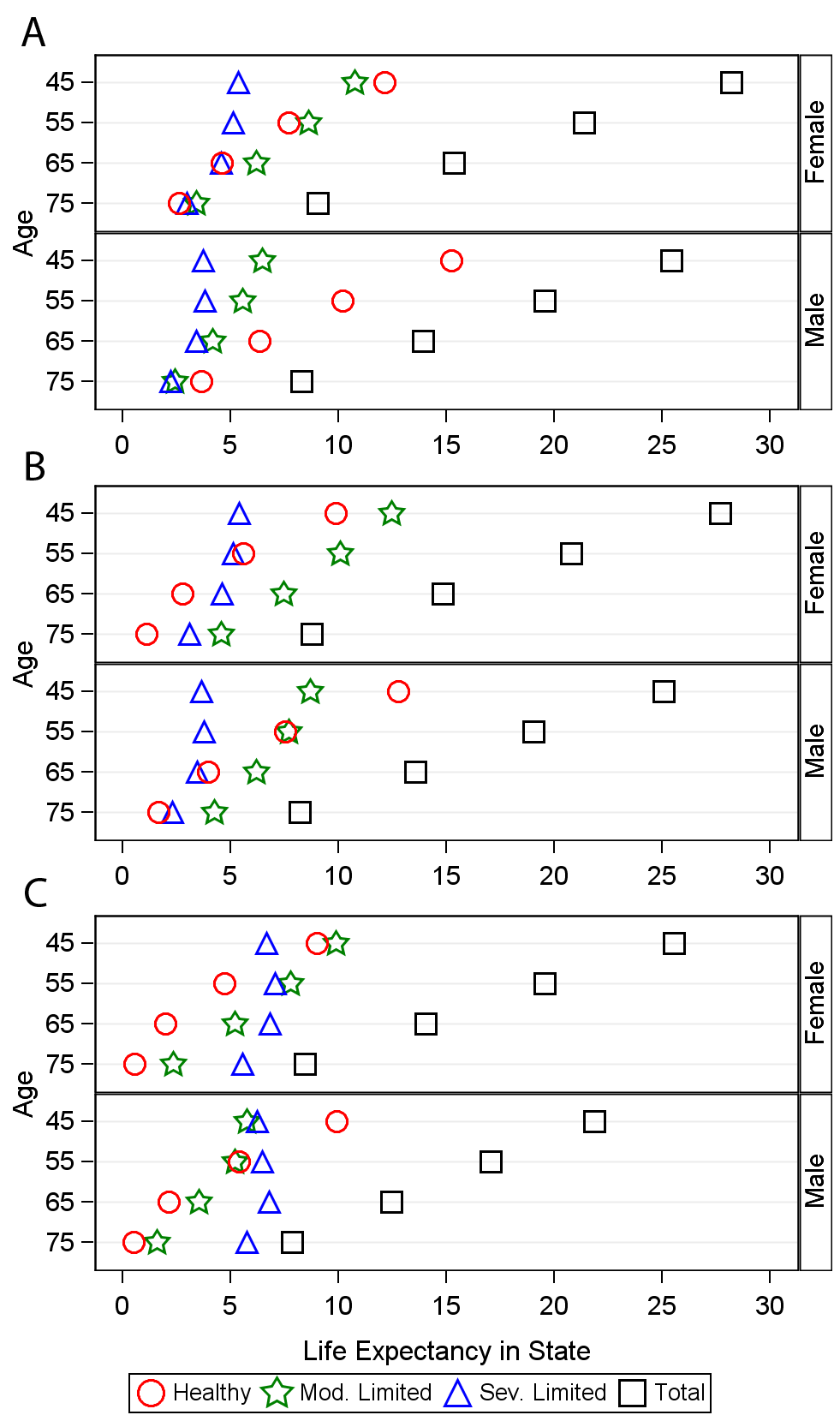

Supplement: Figure S4 — Average number of years of active, moderately limited, and severely limited life, and total life expectancy, conditional on initial disability status. The graphs in this figure show a comparison between the number of years an average individual will spend in healthy, moderately limited, and severely limited life at age 45, 55, 65, and 75 y, conditional on initial disability status (i.e., disability status at age 45, 55, 65, or 75 y for the portion of the simulated synthetic cohort is initially healthy [A], moderately limited [B], or severely limited [C]). As in Figure 4, the markers represent the overall distribution of remaining life-years spent in each state, not the ordering of these life-years; individuals in our analysis can recover and relapse between disability states, so not all years of limitation are spent at the end of life. Differences in overall LE between (A), which conditions on all individuals being healthy at age 45 y, and Figure 4, in which members of the synthetic cohort have the full empirically observed distribution of disability states as shown in Table S4, are relatively small for cohorts beginning at age 45 or 55 y. This is due to two facts: first, at these ages a large fraction of the MLSFH population, and thus the initial health states of the simulated cohorts in Figure 4, is in the healthy state (see Table S4), and, second, at these relatively young ages, the probability of recovering from a moderately limited or severely limited state is relatively high (Figure 3). Therefore, an initial moderately—and with lower probability also a severely—limited state is likely to be transient. At older ages (i.e., a starting age of 65 or 75 y for the synthetic cohort), the differences between Figure 4 and (A) increase as more individuals in Figure 4 enter the synthetic cohort with moderate or severe limitations, and these disabilities become increasingly persistent at older ages (Table S4; Figure 3). Hence, conditioning on a healthy initial state in th [file pmed.1001435.s004.tif]

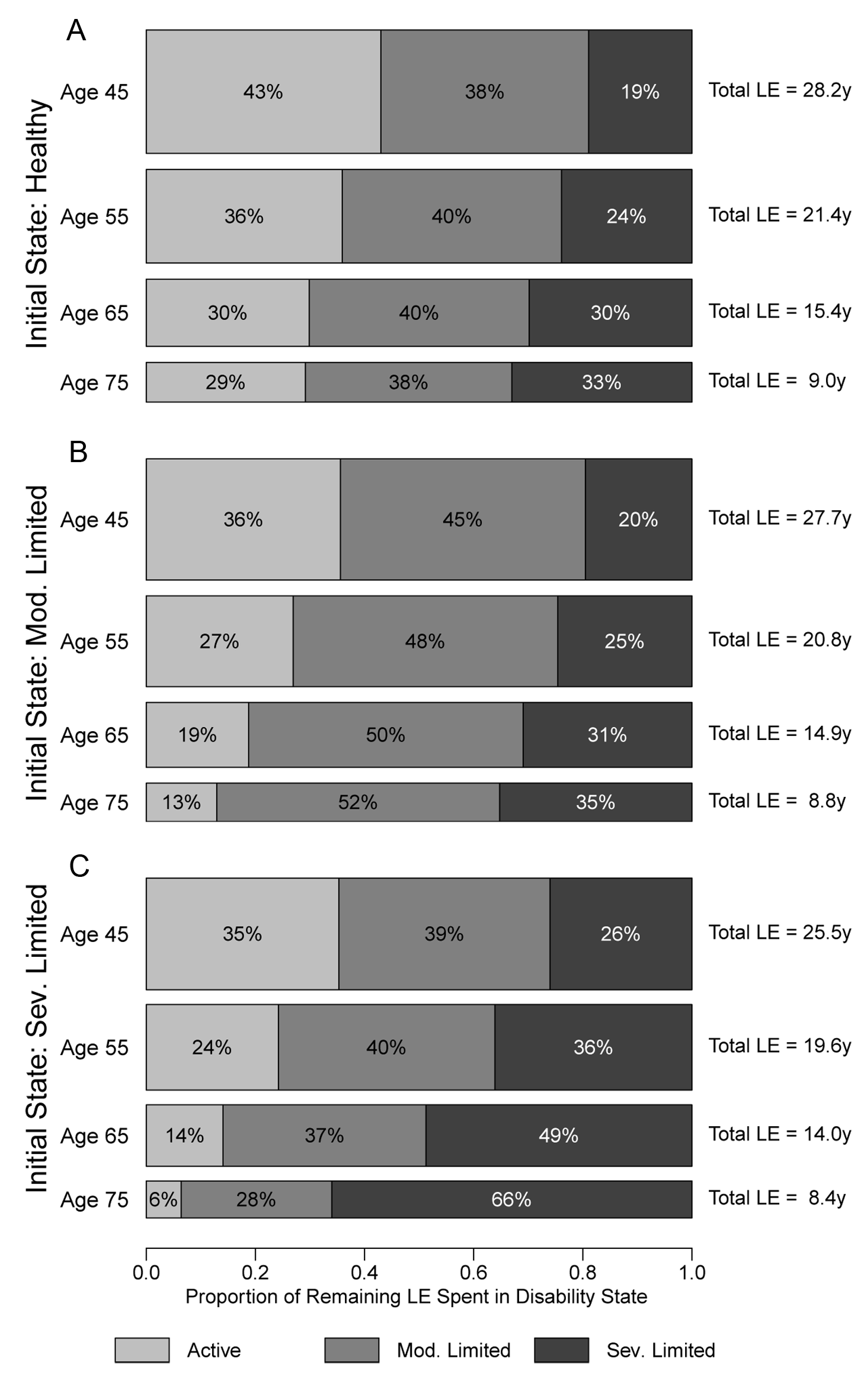

Supplement: Figure S5 — Women: distribution of remaining life expectancy by disability state (healthy, moderately limited, severely limited), conditional on initial disability status. This figure shows the proportion of remaining life an average individual will spend in healthy, moderately limited, and severely limited life at age 45, 55, 65, and 75 y, conditional on the initial disability status for individuals in the synthetic cohort being healthy (A), moderately limited (B), or severely limited (C). The height and area of each bar is proportional to the overall remaining LE of the synthetic cohorts with initial ages of 45, 55, 65, and 75 y, and the differently shaded areas represent the distribution of the remaining LE across the three disability states: healthy, moderately limited, and severely limited. The bars do not necessarily reflect the ordering of these life-years by disability states as individuals in our analysis can recover and relapse between disability states, so not all years of limitation are spent at the end of life. (TIF) [file pmed.1001435.s005.tif]

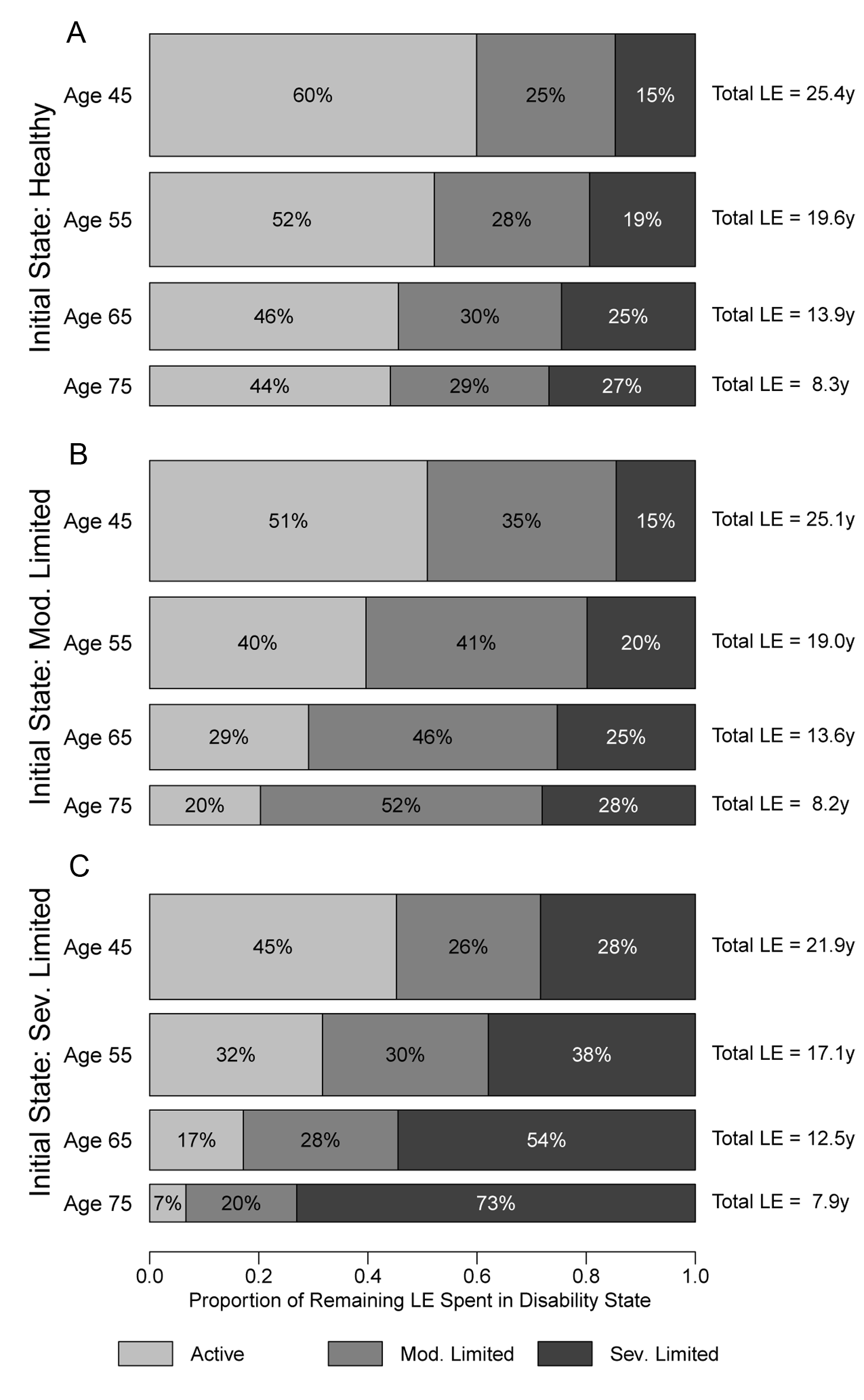

Supplement: Figure S6 — Men: distribution of remaining life expectancy by disability state (healthy, moderately limited, severely limited), conditional on initial disability status. As in Figure S5, but for men. (TIF) [file pmed.1001435.s006.tif]
