# Supplementary material for: Disability Transitions and Health Expectancies among Adults 45 Years and Older in Malawi: A Cohort-Based Model
Source: PLoS Med. 2013 May 7;10(5):e1001435. doi: 10.1371/journal.pmed.1001435 (PMC3646719; doi:10.1371/journal.pmed.1001435)
Supplement: Table S1 — Descriptive statistics for the 2010 MLSFH study population, and comparison of the MLSFH and the IHS3 (rural) sample characteristics. (PDF) [file pmed.1001435.s007.pdf]

Table S1: Descriptive statistics for the 2010 MLSFH study population, and comparison of the MLSFH and the IHS3 (rural) sample characteristics

	45–64				65+			
	MLSFH 2010		IHS3 2010–11		MLSFH 2010		IHS3 2010–11	
	N	%	N	%	N	%	N	%
Demographic and Socioeconomic Characteristics								
Male	327	44.7%	1,924	48.1%	151	46.9%	785	43.2%
Any education	522	71.4%	2,483	62.1%	198	61.5%	745	41.0%
Married	605	83.4%	3,071	76.8%	192	60.8%	914	50.3%
Religion								
Christian	447	61.2%	3,319	83.0%	184	60.5%	1,418	78.0%
Muslim	156	21.4%	441	11.0%	82	27.0%	241	13.3%
Other	134	18.4%	241	6.0%	47	15.5%	158	8.7%
Metal/tile roof	184	25.4%	1,253	31.3%	85	26.9%	537	29.6%
Health Indicators								
Functional limitations and disability state								
Moderate Limitation	239	32.7%	–	–	142	44.1%	–	–
Severe Limitation	64	8.8%	–	–	93	28.9%	–	–
ADL disabled	–	–	783	19.6%	–	–	895	49.3%
Average Age (45+)	61.0		59.8					
Total	731		4,001		322		1,817	

Notes:

(1) IHS3 data description:The Integrated Household Survey is one of the primary instruments implemented by the Government of Malawi through the National Statistical Office (NSO) roughly every 5 years to monitor and evaluate the changing conditions of Malawian households. The IHS data have, among other insights, provided benchmark poverty and vulnerability indicators to foster evidence-based policy formulation and monitor the progress of meeting the Millennium Development Goals (MDGs) as well as the goals listed as part of the Malawi Growth and Development Strategy (MGDS). The Third Integrated Household Survey (IHS3) was conducted by National Statistical Office (NSO) in March 2010-March 2011 [57]. A stratified two-stage sample design was used for the IHS3. The IHS3 sampling frame is based on the listing information and cartography from the 2008 Malawi Population and Housing Census (PHC); includes the three major regions of Malawi, namely North, Center and South; and is stratified into rural and urban strata. The rural subsample of the IHS3, which is used for the above analyses, includes residents from each of the 27 districts of Malawi, except those living in the urban centers of Lilongwe City, Blantyre City, Mzuzu City, and the Municipality of Zomba, and except for residents of the island of Likoma on Lake Malawi. The sampling frame excludes the population living in institutions, such as hospitals, prisons and military barracks.

(2) Health indicators: There are no directly comparable disability/health indicators in the MLSFH and IHS3. Functional limitations and disability states for the MLSFH are defined as follows: respondents who answered "somewhat limited" on either of the two MLSFH SF-12 question about physical limitations are classified as *moderately limited*, and respondents who answered "limited a lot" on either question are classified as *severely limited* (see text for a detailed description. *ADL disabled* in the IHS3 is defined as having difficulty in any one of the following five activities of daily living (ADLs): Seeing, hearing, walking, remembering/concentrating, self-care (bathing/dressing).

(3) Comparisons between the IHS3 and the MLSFH are based on IHS3 and the MLSFH unweighted samples. All differences between the MLSFH and IHS3, except for the proportion male for 45–64 and 65+ and proportion with a metal/tile roof above 65+, are significant ($p < .05$) according to chi-square tests.
