# Supplementary material for: Disability Transitions and Health Expectancies among Adults 45 Years and Older in Malawi: A Cohort-Based Model
Source: PLoS Med. 2013 May 7;10(5):e1001435. doi: 10.1371/journal.pmed.1001435 (PMC3646719; doi:10.1371/journal.pmed.1001435)
Supplement: Table S2 — Regression analyses for associations between disability states and income earned, pain interference with work, dissatisfaction with life, and hours worked on farm. (PDF) [file pmed.1001435.s008.pdf]

Table S2: Regression analyses for associations between disability states and income earned, pain inference with work, dissatisfaction with life, and hours worked on farm

**Logistic Regressions**	Odds Ratio	S.E.	χ^2	Pr > χ^2
Do something to earn income in past week				
Healthy (ref.)	1.00	–	–	–
Mod. Limited	0.95	0.08	0.10	0.76
Sev. Limited	0.49	0.13	7.62	0.01
Pain interfered with work in past 4 weeks				
Healthy (ref.)	1.00	–	–	–
Mod. Limited	4.93	0.08	89.67	<.001
Sev. Limited	11.76	0.15	65.49	<.001
Somewhat/very unsatisfied with life				
Healthy (ref.)	1.00	–	–	–
Mod. Limited	2.55	0.13	13.40	<.001
Sev. Limited	8.88	0.17	40.90	<.001
**OLS Regression**	Estimate	S.E.	t	Pr > $\|t\|$
Hours Worked on Own Farm in Past Week				
Healthy (ref.)	–	–	–	–
Mod. Limited	-0.96	1.20	0.80	0.42
Sev. Limited	-5.32	1.71	3.11	<.001

Notes: Estimates control for age, age2, and gender and account for clustering of standard errors at region and village levels.
