# Supplementary material for: Disability Transitions and Health Expectancies among Adults 45 Years and Older in Malawi: A Cohort-Based Model
Source: PLoS Med. 2013 May 7;10(5):e1001435. doi: 10.1371/journal.pmed.1001435 (PMC3646719; doi:10.1371/journal.pmed.1001435)
Supplement: Table S3 — Distribution of observed transitions between disability states (healthy, moderately limited, severely limited) during 2006–2010 (three MLSFH waves). (PDF) [file pmed.1001435.s009.pdf]

Table S3: Distribution of observed transitions between disability states (healthy/ moderately limited//severely limited) during 2006–2010 (3 MLSFH waves)

Observed distribution among disability states (healthy/moderately limited/ severely limited) and death	Number of transitions	% among all transitions
Healthy at both interviews	682	41.5%
Healthy to moderately limited	283	17.2%
Healthy to severely limited	72	4.4%
Healthy to dead	30	1.8%
Moderately limited to healthy	180	10.9%
Moderately limited at both interviews	171	10.4%
Moderately limited to severely limited	61	3.7%
Moderately limited to dead	29	1.8%
Severely limited to healthy	21	1.3%
Severely limited to moderately limited	43	2.6%
Severely limited at both interviews	55	3.3%
Severely limited to dead	18	1.1%
Total	1645	100%

Notes: See text for definition of disability states; transitions are estimated from all individuals that are observed for at least 2 MLSFH rounds during 2006–10. See also Figure 2 describing the MLSFH study population for these analyses.
