# Supplementary material for: Disability Transitions and Health Expectancies among Adults 45 Years and Older in Malawi: A Cohort-Based Model
Source: PLoS Med. 2013 May 7;10(5):e1001435. doi: 10.1371/journal.pmed.1001435 (PMC3646719; doi:10.1371/journal.pmed.1001435)
Supplement: Table S4 — Initial gender and functional status distribution for the synthetic cohorts ( n = 100,000) used in the microsimulation, as based on the MLSFH. (PDF) [file pmed.1001435.s010.pdf]

Table S4: Initial gender and functional status distribution for the synthetic cohorts (*N* = 100,000) used in the micro-simulation as based on the MLSFH

	Age 45		Age 55		Age 65		Age 75	
	N (MLSFH)	%	*N* (MLSFH)	%	*N* (MLSFH)	%	*N* (MLSFH)	%
Female	673	55.1%	432	49.2%	211	48.2%	131	55.3%
Healthy	427	63.4%	205	47.5%	59	28.0%	31	23.7%
Moderately limited	199	29.6%	174	40.3%	97	46.0%	56	42.7%
Severely limited	47	7.0%	53	12.3%	55	26.1%	44	33.6%
Male	548	44.9%	446	50.8%	227	51.8%	106	44.7%
Healthy	451	82.3%	309	69.3%	123	54.2%	28	26.4%
Moderately limited	86	15.7%	112	25.1%	71	31.3%	46	43.4%
Severely limited	11	2.0%	25	5.6%	33	14.5%	32	30.2%

Notes: The distribution of disability states (healthy/moderately limited/severely limited) is within each gender, while the gender distribution is the fraction male (female) in the synthetic cohort. The synthetic cohort has a size of 100,000, and the *N* reported in the above table pertains to the MLSFH. Gender and functional limitation distributions are estimated from the MLSFH based on 10-year age intervals, that is, the sex and disability apportionment of the microsimulation cohort of 45-year-olds is based on the sex and limitation characteristics of individuals 45–54 in the MLSFH analysis sample.
