# Supplementary material for: Disability Transitions and Health Expectancies among Adults 45 Years and Older in Malawi: A Cohort-Based Model
Source: PLoS Med. 2013 May 7;10(5):e1001435. doi: 10.1371/journal.pmed.1001435 (PMC3646719; doi:10.1371/journal.pmed.1001435)
Supplement: Table S5 — Microsimulation-estimated average remaining life expectancy at ages 45–75 y, by sex, with missing data imputed using multiple imputation by chained equations [67],[68] . (PDF) [file pmed.1001435.s011.pdf]

Table S5: Microsimulation-estimated average remaining life expectancy (LE) at ages 45–75, by sex, with imputed missing data using multiple imputation by chained equations [67, 68]

**Age**	**45**		**55**	
	**LE**	95% CI	**LE**	95% CI
**Female**				
**Life Expectancy**	**25.93**	(23.45–30.77)	**20.24**	(18.16–24.46)
Healthy	**10.36**	(9.23–12.23)	**6.43**	(5.45–7.87)
Mod. Limited	**10.21**	(8.82–12.79)	**8.64**	(7.36–10.84)
Sev. Limited	**5.35**	(4.21–6.83)	**5.17**	(4.01–6.65)
**Male**				
**Life Expectancy**	**23.29**	(21.30–26.58)	**18.38**	(16.60–21.34)
Healthy	**11.64**	(10.53–13.21)	**7.57**	(6.63–8.83)
Mod. Limited	**7.68**	(6.59–9.26)	**6.79**	(5.75–8.35)
Sev. Limited	**3.97**	(3.07–5.24)	**4.01**	(3.05–5.34)

**Age**	**65**		**75**	
	**LE**	95% CI	**LE**	95% CI
**Female**				
**Life Expectancy**	**14.71**	(13.29–19.61)	**9.00**	(8.30–14.63)
Healthy	**3.87**	(3.08–5.18)	**2.27**	(1.66–3.43)
Mod. Limited	**6.25**	(5.23–8.87)	**3.79**	(3.03–6.93)
Sev. Limited	**4.58**	(3.59–6.25)	**2.93**	(2.36–4.53)
**Male**				
**Life Expectancy**	**13.62**	(12.33–16.05)	**8.52**	(7.74–13.60)
Healthy	**4.91**	(4.11–5.98)	**2.75**	(2.05–3.94)
Mod. Limited	**4.98**	(4.11–6.39)	**3.36**	(2.58–6.29)
Sev. Limited	**3.72**	(2.83–4.95)	**2.40**	(1.85–3.76)

Notes: Estimates were obtained from synthetic cohorts of 100,000 45-, 55-, 65-, and 75-year olds created via microsimulation, based on observed transition rates from 2006–2010 MLSFH data. Missing values were imputed using multiple imputation by chained equations [67, 68]. The multiple imputation models included fixed effects for region and village, the full set of SF-12 questions, and data on income, time use, age, ethnicity, religion, gender, number of children, HIV status, and education. 20 datasets with imputed functional status and mortality outcomes for attritors were generated. The multiple imputation analyses in this table provide results that are in close agreement with those obtained without multiple imputation (Table 2), with the primary difference being that the multiple-imputation models estimate a slightly shorter healthy life expectancy for males at age 45 and 55, and a slightly shorter total life expectancy for both sexes at age 45. None of these differences affect the substantive conclusions obtained from our analyses.
