# Supplementary material for: Disability Transitions and Health Expectancies among Adults 45 Years and Older in Malawi: A Cohort-Based Model
Source: PLoS Med. 2013 May 7;10(5):e1001435. doi: 10.1371/journal.pmed.1001435 (PMC3646719; doi:10.1371/journal.pmed.1001435)
Supplement: Table S6 — Microsimulation-estimated average remaining life expectancy at ages 45–75 y, by sex, using a two-level classification of disability (healthy versus limited). (PDF) [file pmed.1001435.s012.pdf]

Table S6: Microsimulation-estimated average remaining life expectancy (LE) at ages 45–75, by sex, using a 2-level classification of disability (healthy vs. limited)

Age	45		55	
	LE	95% CI	LE	95% CI
**Female**				
**Life Expectancy**	**27.47**	(24.54–30.43)	**20.83**	(18.33–22.87)
Healthy	**14.56**	(12.99–15.92)	**8.95**	(7.63–10.10)
Limited	**12.91**	(11.00–14.85)	**11.88**	(10.07–13.82)
**Male**				
**Life Expectancy**	**25.03**	(22.02–28.23)	**19.06**	(16.44–21.34)
Healthy	**16.24**	(14.61–18.32)	**10.65**	(9.24–12.05)
Limited	**8.79**	(6.76–10.77)	**8.41**	(6.70–10.43)
**Age**	**65**		**75**	
	LE	95% CI	LE	95% CI
**Female**				
**Life Expectancy**	**14.61**	(13.01–16.29)	**8.72**	(8.09–9.94)
Healthy	**4.54**	(3.50–5.62)	**2.62**	(1.98–3.64)
Limited	**10.07**	(8.71–11.91)	**6.11**	(5.45–7.61)
**Male**				
**Life Expectancy**	**13.55**	(11.57–15.73)	**8.22**	(7.36–9.49)
Healthy	**6.06**	(4.80–7.21)	**3.50**	(2.74–4.21)
Limited	**7.49**	(6.24–9.36)	**4.72**	(3.97–5.90)

Notes: Estimates were obtained from synthetic cohorts of 100,000 45-, 55-, 65-, and 75-year olds created via microsimulation, based on the observed transition rates from 2006-2010 MLSFH data. Disability classification is based on MLSFH questions (*i*) "Do you have any health problems that limit you in carrying out moderate activities?" and (*ii*) "Do you have any health problems that limit you in carrying out strenuous activities?", with each question providing a list of moderate/strenuous activities and response categories being "not limited", "limited a little" and "limited a lot". Individuals who indicate that they had no functional limitations in either set of activities are classified as *healthy*, those who respond "limited a lot" or "limited a little" on *moderate activities* or "limited a lot" on strenuous activities are classified as *limited*.
