# Supplementary material for: Disability Transitions and Health Expectancies among Adults 45 Years and Older in Malawi: A Cohort-Based Model
Source: PLoS Med. 2013 May 7;10(5):e1001435. doi: 10.1371/journal.pmed.1001435 (PMC3646719; doi:10.1371/journal.pmed.1001435)
Supplement: Table S7 — Microsimulation-estimated average remaining life expectancy at ages 45–75 y, by sex, using a classification of disability based on pain interfering with daily work activities. (PDF) [file pmed.1001435.s013.pdf]

Table S7: Microsimulation-estimated average remaining life expectancy (LE) at ages 45–75, by sex, using a classification of disability based on pain interfering with daily work activities

Age	45		55	
	LE	95% CI	LE	95% CI
**Female**				
**Life Expectancy**	**27.44**	(24.66–38.47)	**22.17**	(18.74–34.91)
Healthy	**9.75**	(8.50–13.21)	**6.40**	(4.83–9.56)
Mod. Limited	**11.80**	(10.11–17.28)	**9.61**	(7.90–15.63)
Sev. Limited	**5.88**	(5.04–8.45)	**6.08**	(4.78–9.41)
**Male**				
**Life Expectancy**	**25.75**	(23.01–31.76)	**20.62**	(17.58–27.28)
Healthy	**13.02**	(11.43–15.37)	**9.07**	(7.47–11.49)
Mod. Limited	**9.24**	(7.65–12.63)	**7.95**	(6.46–10.79)
Sev. Limited	**3.49**	(2.49–5.04)	**3.57**	(2.51–5.43)
**Age**	**65**		**75**	
	LE	95% CI	LE	95% CI
**Female**				
**Life Expectancy**	**14.50**	(13.18–27.81)	**8.55**	(8.12–16.61)
Healthy	**3.40**	(2.49–6.23)	**1.70**	(1.16–2.91)
Mod. Limited	**6.39**	(5.33–13.12)	**3.68**	(2.93–7.42)
Sev. Limited	**4.71**	(4.07–8.39)	**3.18**	(2.82–5.81)
**Male**				
**Life Expectancy**	**14.07**	(12.44–22.00)	**8.32**	(7.61–15.09)
Healthy	**5.51**	(4.44–8.02)	**2.58**	(1.77–4.13)
Mod. Limited	**5.44**	(4.45–9.46)	**3.47**	(2.77–6.98)
Sev. Limited	**3.13**	(2.40–4.91)	**2.26**	(1.71–3.9)

Notes: Estimates were obtained from synthetic cohorts of 100,000 45-, 55-, 65-, and 75-year olds created via microsimulation, based on the observed transition rates from 2006-2010 MLSFH data. Disability classification is based on the MLSFH question "During the past 4 weeks, how much did pain interfere with your normal work (including both work outside the home and housework)?" Individuals who reported no limitations on work caused by pain in the past 4 weeks are categorized as *healthy*, individuals who report "a little bit" or "moderate" limitation are categorized as moderately limited, individuals reporting that pain limited their work activities "quite a bit" or "extremely" are categorized as *severely limited*.
