# Supplementary material for: Disability Transitions and Health Expectancies among Adults 45 Years and Older in Malawi: A Cohort-Based Model
Source: PLoS Med. 2013 May 7;10(5):e1001435. doi: 10.1371/journal.pmed.1001435 (PMC3646719; doi:10.1371/journal.pmed.1001435)
Supplement: Table S8 — MLSFH study population 1998–2010. (PDF) [file pmed.1001435.s014.pdf]

Table S8: MLSFH Study Population 1998–2010

	1998	2001	2004
Number of respondents	2,602	2,548	3,298
Age range (females)	15–49	18-52	15–55
HIV prevalence	—	—	6.4%
Attrition rate	—	14.9%	21.6%
Attrition due to:			
–migration	—	75.6%	58.4%
–mortality	—	13.0%	10.4%
–other factors	—	11.4%	31.2%
	**2006**	**2008**	**2010**
Number of respondents	3,669	4,052	3,790
Age range (females)	17–57	17–92	20–89
HIV prevalence	7.4%	8.9%	—
Attrition rate	19.8%	23.6%	25.6%
Attrition due to:			
–migration	62.5%	57.9%	56.0%
–mortality	6.3%	7.5%	8.8%
–other factors	31.2%	34.6%	35.0%

Notes: Number of respondents is the number of successfully completed MLSFH survey interviews. HIV prevalence in this table is calculated by starting with all HIV positive respondents in the current wave and imputing HIV positive respondents from a prior wave if the HIV positive respondent didn't accept HIV testing in the current wave. HIV positive respondents are significantly more likely to refuse HIV testing in future waves. The attrition rate is the percentage of MLSFH respondents who were successfully interviewed in the previous wave but not interviewed in the current wave. Migration is the most common reason for attrition among MLSFH respondents.
