# Supplementary material for: Disability Transitions and Health Expectancies among Adults 45 Years and Older in Malawi: A Cohort-Based Model
Source: PLoS Med. 2013 May 7;10(5):e1001435. doi: 10.1371/journal.pmed.1001435 (PMC3646719; doi:10.1371/journal.pmed.1001435)
Supplement: Text S1 — MLSFH sampling methods and related relevant data collection procedures. (DOC) [file pmed.1001435.s015.doc]

**Text S1. MLSFH sampling methods and related relevant data collection procedures**

***MLSFH Project Description***

The ***Malawi Longitudinal Study of Families and Health*** (MLSFH) is a collabora­tion of the University of Pennsylvania with the College of Medicine and Chancel­lor College at the University of Malawi. The MLSFH continues earlier research under the ***Malawi Diffusion and Ideational Change Project*** (MDICP). The goal of the MLSFH is to (1) *collect longitudinal data* in rural Malawi that provide a rare record of more than a decade of demographic, socioeconomic and health con­ditions in one of the world’s poorer countries, and (2) *analyze* these data to in­vestigate the multiple inﬂuences that contribute to HIV risks in sexual partner­ships, the variety of ways in which people manage risk within and outside of marriage and other sexual relationships, the possible effects of HIV prevention policies and programs, and the mechanisms through which poor rural individu­als, families, households, and communities cope with the impacts of high AIDS-related morbidity and mortality. For this purpose, the MLSFH has collected lon­gitudinal social science and biomarker data for individuals, families and house­holds on *household structure and family change* (longitudinal household/family ros­ters, marriage and partnership histories), *human capital* (health, schooling, nutri­tional status), *sexual behaviors* (sexual relations, HIV/AIDS risk behaviors and pre­vention strategies), *subjective expectations and well-being* (SF12 module, subjective well-being, HIV risk perceptions, probabilistic expectations about mortality and HIV infection risks), *household production and consumption* (standard of living, time use, migration and remittances, productivity), and *social capital* (social networks, intrafamilial/intergenerational and community transfers, social participation) and *mortality and migration* of MLSFH participants and family members. The MLSFH has conducted repeated *HIV testing and counseling (HTC)*, and has collected data on *anthropometrics* (height, weight and BMI) as well as selected biomarker-based indicators of health (CRP, HDL, LDL and others). Since 2004 the MLSFH data are geocoded. MLSFH data also include linkages between spouses (updated at each round), parent-children linkages, and longitudinal linkages of children listed on the family and household roster. The data collection and research conducted by MLSFH has been approved by the IRB at the University of Pennsylvania and, in Malawi, by the College of Medicine Research Ethics Committee (COMREC) or the National Health Sciences Research Committee (NHSRC). The initial sampling for the MLSFH, and the repeated refreshments of the MLSFH sample are described below, as are some essential ﬁeldwork procedures relevant for this study. The present study is based on the subset of mature adults in the MLSFH, ie., the subset of MLSFH participants who are age 45 and older.

**MLSFH Study Areas:** The MLSFH is based in three districts in rural Malawi that have been the study sites since 1998: Rumphi in the north, Mchinji in the center, and Balaka in the south. In all of these three regions, the primary source of livelihood for MLSFH respondents is subsistence agriculture, augmented with small-scale trade of agricultural products and other goods. Transportation net­works are relatively rudimentary with paved primary roads and generally un­paved secondary roads. Marriage is relatively universal in these rural Malaw­ian regions, with more than 96% of women having ever married by age 25–29, and more than 95% of men having ever married by age 30–34 [1]. While the broad demographic, socioeconomic and epidemiological conditions are fairly sim­ilar across the three MLSFH study regions, and also across other parts of rural Malawi, some noteworthy differences across the MLSFH regions include the fol­lowing. Rumphi District, located in the northern region of the country, follows the patrilineal system of kinship and lineage where residence is ideally patrilocal, inheritance is traced through sons, and parents of a groom pay bridewealth. The northern district, inhabited primarily by Tumbukas, is predominantly Protestant. Mchinji District, located in the central region, follows a less rigid matrilineal sys­tem whereby residence may be matrilocal or patrilocal. The Center is primarily inhabited by Chewas, with almost equal proportions of Catholics and Protestants. Balaka District, which is located in the southern region, is primarily inhabited by Lomwes and Yaos and has the highest proportion of Moslems. The region follows a matrilineal system of kinship and lineage system where residence is ideally ma­trilocal, although it is not uncommon for wives to live at least some period of time in their husband’s village. The Balaka region also exhibits a lower age of sexual debut and larger numbers of lifetime sexual partners than the other MLSFH study regions, and residents tend to be less educated and poorer than those living in the north, leading to higher levels of migration. HIV/AIDS prevalence in the southern region is signiﬁcantly higher than in the northern and central region.

**Initial MLSFH Sample:** The original 1998 MLSFH target sample was 500 ever-married women age 15–49 in each district, plus their husbands (for additional in­formation, see http://malawi.pop.upenn.edu/malawi-documentation-sampling). The sampling strategy adopted for the three districts differed in order to permit comparison with earlier surveys. In Mchinji and Rumphi districts the sample was designed to cover Census Enumeration Areas (CEAs) included in the 1988 Tradi­tional Methods of Child Spacing in Malawi (TMCSM) survey. However, since the TMCSM sampled women regardless of their marital status, the CEAs included in the TMCSM survey had fewer ever-married women than the MDICP target sample of 500 women in each district. Three neighboring CEAs covered by the 1988 survey were thus added to the MLSFH Round 1 sample. In each district a cluster sampling strategy was used in all villages in the selected CEAs. Household lists of those nor­mally resident in those villages were compiled during the week prior to ﬁeldwork, and a sample of eligible women was then randomly selected. Since villages varied in size, sampling fractions were inversely proportional to village populations, such that a higher proportion of eligible women in the smaller villages was sampled. In Balaka district, a somewhat different procedure was followed to allow the evalu­ation of a Community Based Distribution (CBD) initiative that was conducted in this area at the time, following an earlier baseline survey conducted by the German aid agency GTZ with 1098 women and men in 1993. A random subset of 4/7 of the CBD villages and 5/11 of the non-CBD villages from this study were selected as MLSFH study villages. A random 1 in 4 sample of women of reproductive age (15­49) and their husbands was then drawn from these villages to yield a target sample of 500 women and their husbands. To further increase the number of MLSFH re­spondents who participated in the 1993 GTZ survey, an additional 260 women and 125 men were randomly drawn from the GTZ sampling lists (divided equally be­tween the CBD and non-CBD areas) and enrolled in the MLSFH. In total, across all three regions, the MLSFH Round 1 in 1998 enrolled a sample of slightly more than 1,500 ever-married women aged 15–49 and close to 1,100 of their spouses residing in about 120 study villages (Table S8).

**MLSFH Respondent follow-up, migration and vital status:** The MLSFH re­turned to the study areas in 2001, 2004, 2006, 2008 and 2010 to reinterview the MLSFH study population. For this purpose, the MLSFH maintained a respon­dent database that contained previously collected identifying information for each respondent (respondents name, compound name, village name and GPS coordi­nates, etc.). Using this existing identifying information, MLSFH interviewers at­tempted to contact and reinterview MLSFH participants in each of the follow-up years. If MLSFH participants were absent at the ﬁrst interviewer visit, up to two additional follow-up visits were made. Except for a migration follow-up study in 2007, which is not part of the present analyses, MLSFH respondents were not followed if they had migrated outside of the MLSFH study villages. However, they remained in the MLSFH sampling frame, and were interviewed at subsequent MLSFH waves if they returned to the MLSFH study villages (as is common since a signiﬁcant amount of migration is labor-related and thus temporary). On average, the MLSFH succeeded in re-interviewing between about 75–85% of the respon­dents interviewed at the previous MLSFH waves (Supplemental Materials Table S8). When a MLSFH participant could not be found and contacted for a MLSFH follow-up interview, the MLSFH conducted a short interview with family mem­bers and/or neighbors to obtain essential information about the vital status and migration of the MLSFH respondent. Based on this information, the respondent’s status in the MLSFH was recorded as classiﬁed as *dead*, *migrated*, *refused*, *hospital­ized*, *temporarily absent*, *other*, and *unknown*. Conditional on successfully contacting a MLSFH respondents, refusals to participation in the MLSFH have been very low across all MLSFH waves (< 3% up to 2008, and < 5% in 2010).

**MLSFH Sample Additions:** Additions to the MLSFH have occurred primar­ily through three mechanisms: new spouses, the 2004 adolescent sample, and the 2008 parent sample. We discuss these three mechanisms in turn. ***New spouses:*** The initial MLSFH sample in 1998 included 1,541 ever-married women aged 15–49 and their spouses. Up to the 2004 round of data collection, the MLSFH attempted to re-interview all of these initial MLSFH respondents and their current spouses; that is, if a MLSFH respondent divorced and remarried, or in the case of polyga­mous men, added an additional wife, the MLSFH added the current wife (all cur­rent wives) of the initial MLSFH participants. However, spouses who were not part of the initial MLSFH sample were not followed and retained in the MLSFH if they divorced or their spouses died. Starting with the 2006 MLSFH, the study retained all MLSFH study participants; that is, from 2006 onward, once an indi­vidual was interviewed for the MLSFH once, for instance after being enrolled as a new spouse, the MLSFH made an attempt to re-interview the respondent at all subsequent waves. ***2004 Adolescent Sample:*** In 2004, to compensate for the aging of the initial MLSFH sample and the underrepresentation of unmarried individ­uals at adolescent and young adult ages, the MLSFH added an adolescent sam­ple in 2004 (*N* = 998). For this purpose, two household rosters were collected in each sampled community as part of the 2004 MLSFH data collection. The ﬁrst was collected from all households in the sampled villages—that is, MLSFH and non-MLSFH households—during a household listing interview in which all members of all households in the MLSFH were enumerated along with basic demographic characteristics. The second household roster was incorporated into the primary MLSFH survey instrument administered to all female MLSFH participants to enu­merate all eligible adolescents who are part of existing MLSFH households. To allow for intergenerational analyses, all adolescents aged 15–25 listed as members of the existing MLSFH households and residing in the MLSFH study villages were enrolled into the MLSFH adolescent sample, constituting about 1/3 of the total *N* of the adolescent sample. The remaining members of the MLSFH adolescent sample were selected from the household listing conducted for non-MLSFH house­holds using a age-stratiﬁed sampling strategy that adjusted for the differential ages at marriage between gender and MLSFH study regions (for additional informa­tion, see http://malawi.pop.upenn.edu/malawi-documentation-sampling. ***2008 MLSFH Parent Sample:*** To increase the suitability of the MLSFH to study intergen­erational aspects and the health of older individuals in Malawi, a parent sample was added to the MLSFH in 2008. This new sample of parents of MLSFH respon­dents was drawn from family listings from MLSFH respondents in 2006 (because of the respondents’ young age, parents of MLSFH respondents in the 2004 adolescent sample were not included). All living biological parents who resided in the same village as the respondent were included in the 2008 MLSFH new sample of par­ents. Based on this approach, parents of MLSFH respondents living in the MLSFH study villages were added to the 2008 MLSFH sample (*N* = 549). As a result of this enrolling older individuals who were not necessarily captured by the earlier MLSFH sampling frame, the age range covered by the MLSFH was substantially extended by (Supplemental Materials Table S8). Among approximately 3,800 re­spondents interviewed in the 2010 MLSFH, 44.1% were from the original MLSFH sample drawn in 1998, 19.5% were from the 2004 adolescent sample, 12.5% from the 2008 parent sample, and the remainder (23.9%) were new spouses that have been added during 2001–2010.

**Comparisons of the MLSFH with national representative samples:** While the initial sampling strategy of the MLSFH was not designed to be representa­tive of the national population of rural Malawi, the initial sample characteristics closely matched the characteristics of the rural population of the 1996 Malawi De­mographic and Health Survey (DHS) [2]. After three rounds of longitudinal data collection, despite attrition and the enrollment of new subjects, the 2004 MLSFH sample continues to be in close agreement in observable characteristics with the nationally-representative 2004 Malawi DHS (rural sub-population) [3]. After three additional rounds of MLSFH follow-up, the 2010 MLSFH mature adult population— i.e., the study population that is used for the analyses here—continues to closely match the rural subsample in the national-representative IHS3 (Table S1) in key observable characteristics.

***References***

1. Malawi National Statistical Office, ICF Macro (2011) Malawi Demographic and Health Survey 2010: final report. Calverton (Maryland): ICF Macro. Available: http://www.measuredhs.com/publications/publication-fr247-dhs-final-reports.cfm. Accessed 25 March 2013.

2. Watkins S, Behrman JR, Kohler HP, Zulu EM (2003) Introduction to “research on demographic aspects of HIV/AIDS in rural Africa”. Demogr Res Special Collection 1: 1-30.

3. Anglewicz P, Adams J, Obare F, Kohler HP, Watkins S (2009) The Malawi Diffusion and Ideational Change Project 2004–06: data collection, data quality and analyses of attrition. Demogr Res 20: 503-540.
